# Bounded Wang tilings with integer programming and graph-based heuristics

**DOI:** 10.1038/s41598-023-31786-3

**Published:** 2023-03-24

**Authors:** Marek Tyburec, Jan Zeman

**Affiliations:** 1 Department of Mechanics, Faculty of Civil Engineering, Czech Technical University in Prague, Thákurova 7, 16000 Prague 6, Czech Republic; 2 Department of Decision-Making Theory, Institute of Information Theory and Automation, Czech Academy of Sciences, Pod Vodárenskou věží 4, 18200 Prague 8, Czech Republic

**Keywords:** Computational science, Computer science

## Abstract

Wang tiles enable efficient pattern compression while avoiding the periodicity in tile distribution via programmable matching rules. However, most research in Wang tilings has considered tiling the infinite plane. Motivated by emerging applications in materials engineering, we consider the bounded version of the tiling problem and offer four integer programming formulations to construct valid or nearly-valid Wang tilings: a decision, maximum-rectangular tiling, maximum cover, and maximum adjacency constraint satisfaction formulations. To facilitate a finer control over the resulting tilings, we extend these programs with tile-based, color-based, packing, and variable-sized periodic constraints. Furthermore, we introduce an efficient heuristic algorithm for the maximum-cover variant based on the shortest path search in directed acyclic graphs and derive simple modifications to provide a 1/2 approximation guarantee for arbitrary tile sets, and a 2/3 guarantee for tile sets with cyclic transducers. Finally, we benchmark the performance of the integer programming formulations and of the heuristic algorithms showing that the heuristics provide very competitive outputs in a fraction of time. As a by-product, we reveal errors in two well-known aperiodic tile sets: the Knuth tile set contains a tile unusable in two-way infinite tilings, and the Lagae corner tile set is not aperiodic.

## Introduction

Wang tiles, non-rotatable unit squares with colored edges, constitute a formalism introduced by Wang^1^ to visualize the $\forall \exists \forall$ decidability problem of predicate calculus, asking for a general algorithm that decides emptiness of the satisfiable set of all logical formulas of the form “for all *x* there is a *y* such that for all *z*...” followed by a logical combination of predicates without quantifiers. Formulating an equivalent domino problem, Wang considered an infinite number of copies of an arbitrary set of Wang tiles and investigated whether there exists a simply-connected valid tiling of the infinite plane. Moreover, he conjectured^2^ that only the tile sets that form a torus, i.e., cover a periodic simply-connected rectangular domain with identical coloring of the opposite edges, generate infinite valid tilings. Berger^3^ disproved the conjecture by finding a tile set that covers the infinite plane aperiodically by exploiting Kahr’s reduction of the Turing halting problem^4,5^ to the origin-constrained domino problem^6^. Hence, the domino problem was proven to be *undecidable* and, consequently, no general finite algorithm for producing infinite valid tilings exists.

Far less attention has been paid to the finite version of the domino problem, *bounded tiling*, i.e., searching for a fixed-sized valid tiling generated by an arbitrary tile set. In contrast to the infinite variant, the bounded tiling is $\mathscr{N}\mathscr{P}$-complete in general, and thus decidable, e.g.,^7^ or^8^, Theorem 7.2.1], so that finite-time algorithms can be developed. However, most of the available approaches exploit specific properties of particular tile sets^9–12^ or address the tile packing problem for edge-matching puzzles, in which all tiles from the set are placed exactly once^13–16^. Another closely related problem emerges in tiling with polyominoes^17^.

In this work, we investigate the bounded Wang tiling problem in its full generality. To this goal, we first survey the most significant *aperiodic* tile sets in “Aperiodic tile sets” section and applications of Wang tiles in “Applications of Wang tiles” section. In “Wang tiling generation algorithms” section, we list available algorithms for generation of Wang tilings. Finally, our aims and contributions appear summarized in “Aims and novelty” section.

### Aperiodic tile sets

**Figure 1:**
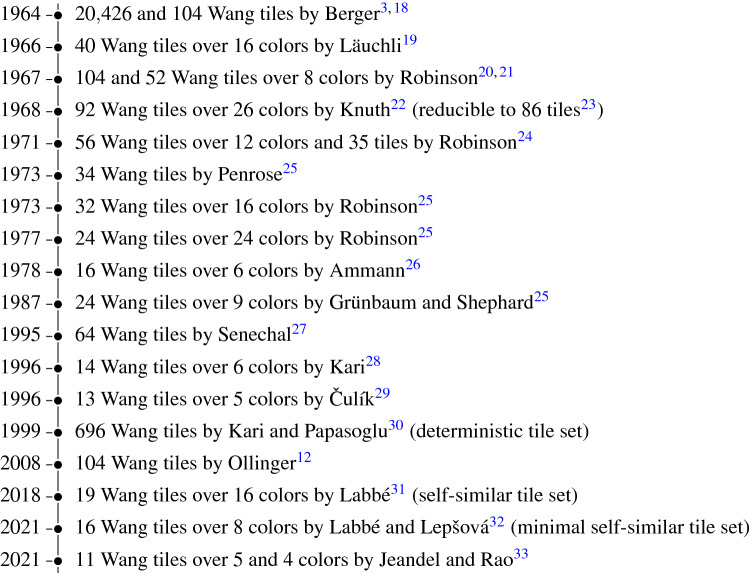
List of aperiodic Wang tile sets.

The originally unexpected property of Wang tile sets—aperiodicity—resulted in a long-term competition among scientists in mathematical logic, computer science, discrete mathematics, and even recreational mathematicians to find the aperiodic tile set of the minimum cardinality^25^, Chapter 11]. Starting from the Berger tile set containing 20, 426 tiles in 1964^3,18^, it took almost 50 years until the two sets of 11 tiles were found and proved to be minimal^33^; see Fig. 1 for a graphical overview of the selected historical developments.

In 1966, Läuchli sent to Wang an aperiodic set of 40 tiles over 16 colors, but it remained unpublished until 1975^19^. Meanwhile, unaware of the Läuchli’s result, Knuth^22^ simplified Berger’s set to 92 tiles over 26 colors; and Robinson developed sets of 104 and 52 tiles over 8 colors in 1967^21^, of 56 tiles over 12 colors in 1971^24^, and anticipated an existence of a set of 35 tiles^24^.

In 1973, Penrose developed a new approach based on kite and dart tiling, leading to a set of 34 tiles^25^. Robinson, being in contact with Penrose, modified Penrose’s approach to reach a reduced set of 32 tiles over 16 colors^25^. Using the same technique together with Penrose rhombs tiling, Grünbaum and Shephard^25^ obtained a set of 24 tiles over 9 colors in 1987.

Another two tile sets were discovered by Ammann. In 1978, he used the so-called Ammann bars to reach 16 tiles over 6 colors^26^. Building on the Ammann’s A2 tiling^25^, Robinson obtained a set of 24 tiles over 24 colors in 1977^25^.

Subsequent size reduction of the smallest aperiodic set occurred in 1996, when Kari^28^ developed a new method based on Mealy machines multiplying Beatty sequences and presented a set of 14 tiles over 6 colors. Čulík^29^, using the same approach, reduced the set even further to 13 tiles over 5 colors.

The search for the minimal aperiodic set was concluded by Jeandel and Rao^33^, who used an enumeration approach to find aperiodic sets of 11 tiles over 4 and 5 colors and proved non-existence of an aperiodic set either containing 10 or fewer tiles or labeled by less than 4 colors.

In addition to the original Wang tiles, in 2006, Lagae and Dutré^34^ described a subset of the Wang tiles, the *corner* tiles (we refer to the Appendix section of the supplementary material for their relation to edge-based Wang tiles), with the adjacency rules stored in the colored corners instead of the edges. In the same year, they constructed multiple aperiodic sets of corner tiles^35^, out of which the set of 44 corner tiles over 6 colors was the smallest one. The set was further simplified by Nurmi^36^ to 30 corner tiles over 6 colors and both were claimed to be aperiodic.

### Applications of Wang tiles

Thanks to the property of particular tile sets to generate aperiodic tilings, Wang tiles gained interest among several disciplines. Building on the original purpose of Wang tiles, proofs in the first-order logic^2^, they were also used in cellular automata theory^37^, topology, group theory^38^, and symbolic dynamical systems^39^.

In computer graphics, Stam^40^ adopted Wang tiles to generate aperiodic textures by assigning a pattern to each tile and assembled seamless continuous textures through valid tilings. After Cohen et al.^9^ recognized that stochastic nonperiodic tilings are easier to handle computationally and provide larger degrees of freedom, the Wang-tile-based approach to generating seamless textures became popular, also including the generation of point patterns and volumes^41^ with predefined statistical properties^9,42^.

In science, Wang tiles and other related aperiodic tilings served as the key tool for understanding the 5-fold symmetry of electron diffraction patterns of quasicrystals^27,43^. Another application at the nanoscale involved molecular DNA-based realization of Wang tiles, introduced by Winfree et al.^44^, which provided a self-assembly of biological nanostructures into aperiodic patterns. The self-assembly process of DNA Wang tiles also powered custom DNA-based computations^45^, fueled by Turing completeness of Wang tiles^3,19^.

Beyond the nanoscale, Wang tiles have also been used for efficient compression^46^ and reconstruction^47^ of microstructures, generalizing the traditional Periodic-Unit-Cell homogenization-based framework to the nonperiodic setting^48^. Consequently, numerical analyses of random heterogeneous materials can be accelerated by exploiting the multiple tile occurrences in the physical domain^49,50^. For an inverse problem of designing modular, nonperiodic structures or materials, we have developed a bilevel approach to optimize truss modules based on the corner Wang tiling formalism^51^ and a clustering-based method for designing modular structures and mechanisms with continuum topology optimization^52^. In these works, the colorings of the Wang tile edges encode the information how modules can be assembled to maintain a mechanically-compatible design. Finally, Jílek et al.^53,54^ developed a centimeter-scale self-assembly procedure building on the Wang tiling formalism.

### Wang tiling generation algorithms

To the best of our knowledge, no general approaches to solving the bounded tiling problem have been reported in the literature; the only available results are specific to single families of tile sets^9–12^, or consider infinite thin strips^33^. In what follows, we describe the gist of three tiling algorithms: substitution-based, stochastic, and transducer-based.

#### Substitution-based tiling algorithm

Given a tile set $\mathscr {T}$, substitution is a map $S: \mathscr {T} \mapsto \mathfrak {T}$ that assigns a tiling $\mathfrak {T}_k$ to each tile $k \in \mathscr {T}$; we refer the reader to “Notation and preliminaries” section for the definitions. Consequently, arbitrary-sized tilings follow from a placed tile *k* and a recursively applied substitution rule^12^. Hence, the tiling “grows” iteratively. Clearly, such a procedure has a low complexity, but only very specific tile sets allow for such substitution rule that generates valid tilings.

#### Stochastic tiling algorithms

In computer graphics, Wang tiles have mostly been used for generating visually appealing yet compressed textures. For this, it is essential to generate these nonperiodic patterns quickly, which is best achieved with stochastic tile sets—usually containing all combinations of edge labels for a given number of colors. For example, in the stochastic tiling algorithm^9^, the tiling is generated row-wise, such that the neighbor of any tile that has already been placed can always be selected from at least two tiles at random. This approach was further extended towards the hash-based direct stochastic tiling algorithm^11^. Note that stochastic algorithms enable straightforward enforcement of several tile- or edge-based constraints.

#### Transducer-based tiling algorithm

The transducer-based tiling algorithm^33^ builds on the fact that the 1D domino problem is decidable and can be solved in a polynomial time because the bi-infinite path is formed by an arbitrary cycle in transducer graphs, see “Notation and preliminaries” section for clarification. To generate valid tilings of multiple rows, the product of several transducers must be computed. Hence, we must enumerate all feasible valid tilings for a requested height and unit width, and then find a path of the given length in the transducer graph of the just-formed tile set. Obviously, this approach works well for tiling thin strips; however, it is impractical for larger nearly-square domains.

### Aims and novelty

In this contribution, we consider the bounded Wang tiling in its general form, thereby allowing arbitrary tile sets and control over the resulting tilings. As follows from the above state-of-the-art survey, no such method has been published yet.

We believe that development of such algorithms is important from multiple reasons. First, we have already investigated modeling and optimization of non-periodic and stochastic microstructures with Wang tilings^46,47,49–51,55^. We hope that the extension of our methods to more general tile sets would enable characterizing a broader class of non-periodic conventional materials and meta-materials^48,56–58^ and thus also improve upon the performance of optimized designs. Due to their Turing completeness^3,19^, Wang tiles might also potentially bridge the fields of meta-materials^56^ and mechanical computing^59^. In this direction, generation of bounded tiling represents a design of particular finite automaton from the (material) states defined by the design of individual tiles.

Apart from emerging applications in materials engineering, we believe that developing a unified methodology is of independent interest, e.g., for the verification of the results available in the literature. Here we justify this claim by finding two errors in well-established aperiodic tile sets.

To do this, we first provide the necessary definitions in “Notation and preliminaries” section to make the manuscript self-contained. The subsequent part is devoted to four integer programming formulations for generation of valid tilings: decision variant in “Rectangular valid tiling” section, maximum rectangular valid tiling in “Maximum rectangular valid tiling” section, maximum-cover in “Maximum cover” section, and maximum adjacency constraint satisfaction in “Maximum adjacency constraints satisfaction” section. To allow for a finer control over the resulting tilings, we also include simple extensions to prescribe tile- and color-based boundary conditions, periodic constraints, and the tile-packing constraint in “Extensions” section.

Due to the complexity of the proposed formulations, in “Heuristic algorithm for the maximum cover tiling problem” section we propose a heuristic graph-based algorithm to tackle the maximum-cover optimization variant from “Maximum cover” section. The developed algorithm relies on solutions to shortest path problems in directed acyclic graphs, hence possesses a low asymptotic complexity. Further, we show that a slight modification maintains an approximation ratio of 2/3 for the tile sets whose transducer graphs are cyclic.

“Results” section collects results from the computational assessment of the integer programming formulations (“Integer programming formulations” section) and heuristics (“Heuristic algorithms” section), and on the benchmarking of the periodic tile packing formulation against the algorithm of Lagae and Dutré^14^ (“Periodic tile packing problem” section). We close the section with two surprising observations found with integer programming for two well-known aperiodic tile sets: the Knuth^22^ tile set of 92 tiles contains a tile unusable in infinite simply-connected valid tilings, “Unusable tile in the Knuth tile set” section, and the Lagae et al.^35^ tile set of 44 corner tiles is not aperiodic, “Periodicity of the Lagae corner tile set” section. We summarize our results in “Conclusions” section.

## Notation and preliminaries

**Figure 2:**
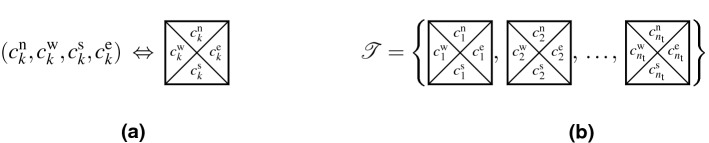
Graphical representation of (**a**) a Wang tile *k*, and of (**b**) a tile set $\mathscr {T}$.

Considering a finite set of *color codes* $\mathscr {C} = \{1, 2,\dots , n_\mathrm{c}\} \subset \mathbb {N}$, the *(Wang) tile* *k* is a quadruple of the color codes $(c^\mathrm{n}_{k}, c^\mathrm{w}_{k}, c^\mathrm{s}_{k}, c^\mathrm{e}_{k})$, with $c^\mathrm{n}_{k}, c^\mathrm{w}_{k}, c^\mathrm{s}_{k}$, and $c^\mathrm{e}_{k} \in \mathscr {C}$ standing for the color codes of the north, west, south, and east edge of the tile *k*, respectively. Tiles can, therefore, be represented graphically as non-rotatable squares shown in Fig. 2a. Without loss of generality, we further consider these squares to be of the unit size.

A *tile set* $\mathscr {T}$ represents a finite collection of $n_\mathrm{t}$ tiles, see Fig. 2b. When $\forall (c^\mathrm{n},c^\mathrm{w},c^\mathrm{s},c^\mathrm{e}) \in \mathscr {C}^4: (c^\mathrm{n},c^\mathrm{w},c^\mathrm{s},c^\mathrm{e}) \in \mathscr {T}$, we call the tile set *complete*.

**Figure 3:**
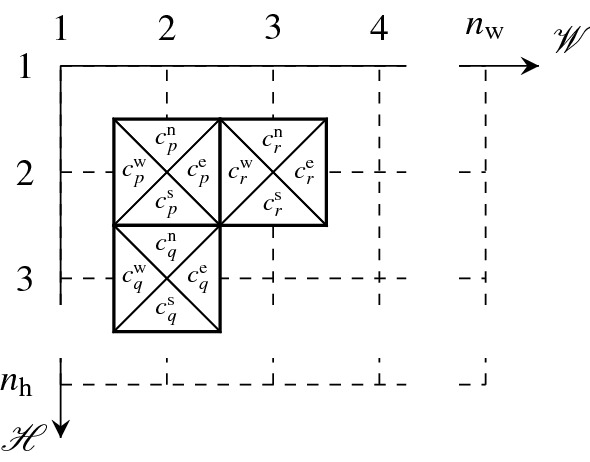
Color matching among tiles *p*, *q*, and $r \in \mathscr {T}$.

Using the notation $\tilde{\bullet } = \bullet \cap \mathbb {Z}^2$ to denote an intersection of the set $\bullet$ with the integer lattice points, *tiling* $\mathfrak {T}^\mathscr {A}$ of a bounded domain $\mathscr {A} \in \mathbb {R}^2$ is an arrangement of copies of the tiles from $\mathscr {T}$ such that the tiles are placed at $\tilde{\mathscr {A}}$, and cover the entire domain $\mathscr {A}$, cf. Fig. 3. More formally, tiling is a mapping $\mathfrak {T}^\mathscr {A}: \tilde{\mathscr {A}} \rightarrow \mathscr {T}$ assigning a single tile $k \in \mathscr {T}$ to every coordinate $(i,j) \in \tilde{\mathscr {A}}$. Consequently, we call tilings $\mathfrak {T}^\mathscr {A}$ *simply connected* iff the domain $\mathscr {A}$ is so.

The tiling $\mathfrak {T}^\mathscr {A}$ is *rectangular* if $\forall i \in \mathscr {H}, \mathscr {H} = \{1,\dots , n_\mathrm{h}\},$ and $\forall j \in \mathscr {W}, \mathscr {W} = \{1,\dots , n_\mathrm{w}\}$, it holds that $(i,j) \in \tilde{\mathscr {A}}$. Here, $\mathscr {H}$ and $\mathscr {W}$ are the sets of the height and width coordinates.

A *valid tiling* (Wang tiling) of $\mathscr {A}$, denoted by $\mathfrak {T}^\mathscr {A}_\mathrm{valid}$, is a tiling with equal color codes at the shared edges between all pairs of adjoining tiles. Therefore, the mapping $\mathfrak {T}^\mathscr {A}_\mathrm{valid}: \tilde{\mathscr {A}} \rightarrow \mathscr {T}$ satisfies, in addition to the requirements for $\mathfrak {T}^\mathscr {A}$, the additional constraints

1a $$\begin{aligned} c^\mathrm{s}_{\mathfrak {T}^\mathscr {A}_\mathrm{valid}(i,j)}&= c^\mathrm{n}_{\mathfrak {T}^\mathscr {A}_\mathrm{valid}(i+1,j)}, \quad \forall (i,j) \in \tilde{\mathscr {A}}: (i+1,j) \in \tilde{\mathscr {A}}, \end{aligned}$$

1b $$\begin{aligned} c^\mathrm{e}_{\mathfrak {T}^\mathscr {A}_\mathrm{valid}(i,j)}&= c^\mathrm{w}_{\mathfrak {T}^\mathscr {A}_\mathrm{valid}(i,j+1)}, \quad \forall (i,j) \in \tilde{\mathscr {A}}: (i,j+1) \in \tilde{\mathscr {A}}, \end{aligned}$$

provided that the axes are oriented accordingly to Fig. 3. If such $\mathfrak {T}^\mathscr {A}_\mathrm{valid}$ exists, we say that the domain $\mathscr {A}$ admits a valid $\mathscr {T}$-tiling, or that it is *tileable* by $\mathscr {T}$.

Consider that $\mathscr {B}\subseteq \mathscr {A}$ and $\mathscr {B}_{{\text {max rect}}} \subseteq \mathscr {A}$ are simply connected, rectangular, and $\mathscr {T}$-tileable. Then, the *maximum rectangular valid tiling* $\mathfrak {T}^\mathscr {A}_{\textrm{v}, \text {max rect}}$ is a valid tiling of the domain $\mathscr {B}_{\text {max rect}}$, where $\{\mathscr {B}_{\text {max rect}} \subseteq \mathscr {A}, \forall \mathscr {B} \subseteq \mathscr {A}: |\tilde{\mathscr {B}}_{\text {max rect}} |\ge |\tilde{\mathscr {B}} |\}$. Here, the notation $|\bullet |$ denotes cardinality of the set $\bullet$.

The *maximum cover* $\mathfrak {T}^\mathscr {A}_{\text {v}, {\text {max cov}}}$ is a valid tiling of $\mathscr {B}_{{\text {max cov}}}$, where $\mathscr {B}$ and $\mathscr {B}_{\text {max cov}}$ are arbitrary $\mathscr {T}$-tileable subdomains in $\mathscr {A}$ and $\{\mathscr {B}_{\text {max cov}} \subseteq \mathscr {A}, \forall \mathscr {B} \subseteq \mathscr {A}: |\tilde{\mathscr {B}}_{\text {max cov}} |\ge |\tilde{\mathscr {B}} |\}$.

A rectangular valid tiling is said to be *periodic*, if the color codes at the opposite sides of the rectangle match. If the valid tiling is not periodic, but the considered tile set allows for at least one periodic rectangular tiling, we call it *nonperiodic*. Finally, if no such periodic pattern exists and the tile set still allows for a valid tiling of the infinite plane, it is referred to as *aperiodic*. Similarly, the tile set $\mathscr {T}$ is *periodic* if it permits periodic valid tilings; and *aperiodic* if all feasible valid tilings are aperiodic.

*Transducer graph*^28^ $G_\mathrm{t,h}$ of the tile set $\mathscr {T}$ is a directed (multi-)graph representation of a Mealy machine without any initial nor terminal state. It consists of $|\mathscr {C}|$ states (graph vertices) and $|\mathscr {T} |$ transitions (directed edges) $\mathscr {E}_\mathrm{h}$, where

2 $$\begin{aligned} \mathscr {E}_\mathrm{h} :=\bigcup _{k \in \mathscr {T}}\left( c_k^\mathrm{w} \xrightarrow {c_k^\mathrm{s}\vert c_k^\mathrm{n}} c_k^\mathrm{e}\right) . \end{aligned}$$

For the *dual transducer graph* $G_\mathrm{t,v}$, composed of the dual Wang tiles^31^ reflecting $\mathscr {T}$ along the major diagonal of the tiles, the edge set is defined as

3 $$\begin{aligned} \mathscr {E}_\mathrm{v} :=\bigcup _{k \in \mathscr {T}}\left( c_k^\mathrm{n} \xrightarrow {c_k^\mathrm{e}\vert c_k^\mathrm{w}} c_k^\mathrm{s}\right) . \end{aligned}$$

**Figure 4:**
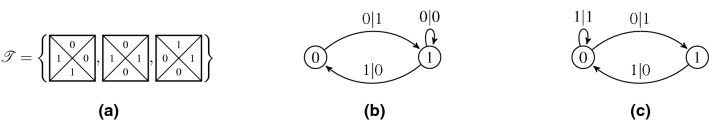
(**b**) Transducer and (**c**) dual transducer graphs of the tile set (**a**).

To illustrate the construction, we include a visual example in Fig. 4.

## Integer programming formulations

In this section, we introduce four integer programming formulations for the generation of valid tilings. The first one, in “Rectangular valid tiling” section, develops a decision variant. In the later sections, we investigate the maximum rectangular tiling (“Maximum rectangular valid tiling” section), maximum cover (“Maximum cover” section), and the maximum adjacency constraints satisfaction (“Maximum adjacency constraints satisfaction” section). Finally, “Extensions” section proposes several extensions to facilitate finer control over the resulting tilings.

### Rectangular valid tiling

Let us now consider the fundamental problem of finding $\mathfrak {T}^\mathscr {A}_\mathrm{v}$ or proving it does not exist. From now on, we restrict $\mathscr {A}$ to be rectangular to simplify notation. However, the presented approach also extends to the general case.

To achieve this, we introduce $\forall (i,j,k) \in \mathscr {H}\times \mathscr {W}\times \mathscr {T}$ a binary decision variable $x_{i,j,k} \in \{0,1\}$ denoting the placement of the tile *k* at the (*i*, *j*) coordinate such that

4 $$\begin{aligned} x_{i,j,k} = \left\{ \begin{array}{c l} 1 &{} \text {iff the tile }k\text { lies at coordinate }(i,j),\\ 0 &{} \text {otherwise.} \end{array}\right. \end{aligned}$$

Consequently, mapping $\mathfrak {T}^\mathscr {A}(i,j)$ is expressed as

5 $$\begin{aligned} \mathfrak {T}^\mathscr {A}(i,j) = \sum _{k \in \mathscr {T}} k x_{i,j,k}, \end{aligned}$$

together with the requirement that every (*i*, *j*) coordinate is occupied by one tile,

6 $$\begin{aligned} \sum _{k \in \mathscr {T}} x_{i,j,k} = 1,\quad \forall (i,j) \in \mathscr {H} \times \mathscr {W}. \end{aligned}$$

Similarly, the color codes of a tile placed at (*i*, *j*) are expressed using the binary variables as

7a $$\begin{aligned} c_{\mathfrak {T}^\mathscr {A}(i,j)}^\mathrm{n}&= \sum _{k \in \mathscr {T}} c_k^\mathrm{n} x_{i,j,k}, \end{aligned}$$

7b $$\begin{aligned} c_{\mathfrak {T}^\mathscr {A}(i,j)}^\mathrm{w}&= \sum _{k \in \mathscr {T}} c_k^\mathrm{w} x_{i,j,k}, \end{aligned}$$

7c $$\begin{aligned} c_{\mathfrak {T}^\mathscr {A}(i,j)}^\mathrm{s}&= \sum _{k \in \mathscr {T}} c_k^\mathrm{s} x_{i,j,k}, \end{aligned}$$

7d $$\begin{aligned} c_{\mathfrak {T}^\mathscr {A}(i,j)}^\mathrm{e}&= \sum _{k \in \mathscr {T}} c_k^\mathrm{e} x_{i,j,k}. \end{aligned}$$

Inserting (7) into (1a) and (1b) leads to the horizontal and vertical adjacency constraints expressed in terms of the decision variables, as

8a $$\begin{aligned} \sum _{k \in \mathscr {T}} c_k^\mathrm{s} x_{i,j,k} - \sum _{k \in \mathscr {T}} c_k^\mathrm{n} x_{i+1,j,k}&= 0, \quad \forall (i,j) \in \mathscr {H}\setminus \{n_\mathrm{h}\}\times \mathscr {W}, \end{aligned}$$

8b $$\begin{aligned} \sum _{k \in \mathscr {T}} c_k^\mathrm{e} x_{i,j,k} - \sum _{k \in \mathscr {T}} c_k^\mathrm{w} x_{i,j+1,k}&= 0, \quad \forall (i,j) \in \mathscr {H}\times \mathscr {W}\setminus \{n_\mathrm{w}\}. \end{aligned}$$

Combining (4), (5), (6), and (8) then provides us with a complete binary linear programming representation of valid tiling $\mathfrak {T}^\mathscr {A}_\mathrm{valid}$.

For computational reasons, it proved to be advantageous to organize the constraints according to the color codes:

9a $$\begin{aligned} \sum _{k \in \mathscr {T}} x_{i,j,k}[c_k^\mathrm{s}=\ell ] - \sum _{k \in \mathscr {T}} x_{i+1, j, k}[c_k^\mathrm{n}=\ell ]&= 0,\quad \forall (i,j,\ell ) \in \mathscr {H}\setminus \{n_\mathrm{h}\} \times \mathscr {W}\times \mathscr {C}, \end{aligned}$$

9b $$\begin{aligned} \sum _{k \in \mathscr {T}} x_{i,j,k}[c_k^\mathrm{e}=\ell ] - \sum _{k \in \mathscr {T}} x_{i, j+1, k}[c_k^\mathrm{w}=\ell ]&= 0,\quad \forall (i,j,\ell ) \in \mathscr {H} \times \mathscr {W}\setminus \{n_\mathrm{w}\} \times \mathscr {C}, \end{aligned}$$

where, in the Iverson notation^60^, $\sum _{k \in \mathscr {T}} x_{i,j,k} \left[ c_k^\mathrm{s} = \ell \right]$ expresses that $x_{i,j,k}$ is added to the sum if and only if $c_k^\mathrm{s} = \ell$.

The constraint (9a) requires that the number of tiles at (*i*, *j*) with the south edge colored by $\ell$ equals to the number of tiles at $(i+1,j)$ with the north edge marked by the same $\ell$, for all $\ell \in \mathscr {C}$. Because of (6), there are either no tiles with the shared edge colored by $\ell$, or there is a single tile at each of the coordinates with its common edge labeled by $\ell$. Analogously to the vertical adjacency constraint, the horizontal constraint (9b) also enforces equality among the number of tiles at (*i*, *j*) with the east edge colored by $\ell$ and the number of tiles at $(i,j+1)$ having the west edge colored by identical $\ell$.

Finally, combining (4), (6), and (9), while noticing that the constraints (6) naturally propagate with the adjacency constraints from the domain boundaries (compare (10d, 10e with (6)), leads to the binary programming formulation

10a $$\begin{aligned} \textrm{find}\;&\textbf{x} \end{aligned}$$

10b $$\begin{aligned} \mathrm {s.t.}\,&\sum _{k \in \mathscr {T}} x_{i,j,k}[c_k^\mathrm{s}=\ell ] - \sum _{k \in \mathscr {T}} x_{i+1, j, k}[c_k^\mathrm{n}=\ell ] = 0,\quad \forall (i,j, \ell ) \in \mathscr {H}\setminus \{n_\mathrm{h}\}\times \mathscr {W}\times \mathscr {C}, \end{aligned}$$

10c $$\begin{aligned}&\sum _{k \in \mathscr {T}} x_{i,j,k}[c_k^\mathrm{e}=\ell ] - \sum _{k \in \mathscr {T}} x_{i, j+1, k}[c_k^\mathrm{w}=\ell ] = 0,\quad \forall (i,j,\ell ) \in \mathscr {H} \times \mathscr {W}\setminus \{n_\mathrm{w}\} \times \mathscr {C}, \end{aligned}$$

10d $$\begin{aligned}&\sum _{k \in \mathscr {T}} x_{i,j,k} = 1, \quad \forall (i,j) \in \{1, n_\mathrm{h}\} \times \mathscr {W}, \end{aligned}$$

10e $$\begin{aligned}&\sum _{k \in \mathscr {T}} x_{i,j,k} = 1, \quad \forall (i,j) \in \mathscr {H} \times \{1, n_\mathrm{w}\}, \end{aligned}$$

10f $$\begin{aligned}&x_{i,j,k} \in \{0,1\}, \quad \forall (i,j,k) \in \mathscr {H} \times \mathscr {W}\times \mathscr {T}, \end{aligned}$$

that provides a complete representation of the bounded tiling problem, i.e., all valid tilings solve the integer program, and conversely, all feasible solutions to (10) are valid tilings. Moreover, observe that the problem consists of two totally unimodular constraints if considered independently: (10c, 10e) representing row tilings, and (10b, 10d) being column tilings. When considered simultaneously, the resulting problem becomes $\mathscr{N}\mathscr{P}$-complete^7,8^.

### Maximum rectangular valid tiling

When a solution to (10) cannot be found in an acceptable time period or when no such solution exists, one can resort to relaxing the requirement of a valid tiling of $\mathscr {A}$ and search for a valid tiling of the largest rectangular subdomain.

**Figure 5:**
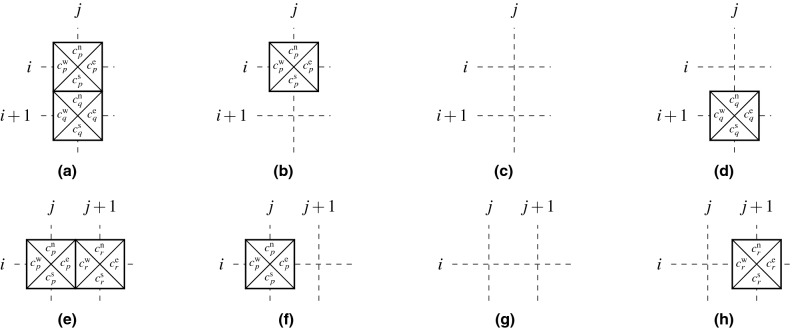
Admissible tile placements (**a**)–(**c**) and (**e**)–(**g**), and forbidden placements (**d**) and (**h**) in the maximum rectangular valid tiling formulation.

Without loss of generality, let us assume that the maximum rectangular valid tiling always contains an anchor tile placed at (1, 1), i.e.,

11 $$\begin{aligned} \sum _{k \in \mathscr {T}} x_{1,1,k} = 1. \end{aligned}$$

On the other hand, all the other coordinates may contain a tile or be empty, thus

12 $$\begin{aligned} \sum _{k \in \mathscr {T}} x_{i,j,k} \le 1, \quad \forall (i,j) \in \tilde{\mathscr {A}} \setminus (1,1). \end{aligned}$$

**Figure 6:**
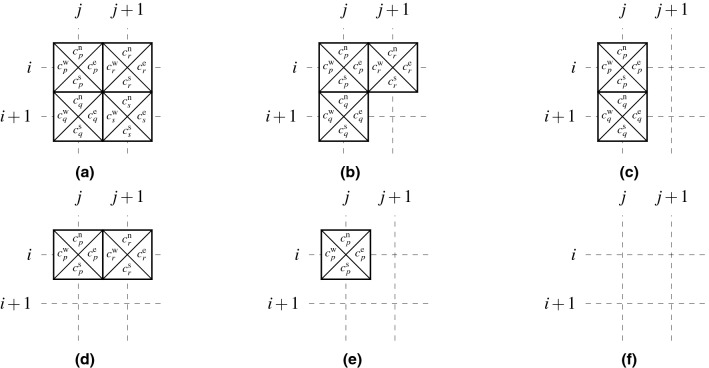
Six possible placements of tiles *p*, *q*, *r*, and *s*. While the combinations (**a**) and (**c**)–(**f**) can appear in rectangular tilings, the combination (**b**) cannot.

Let us now pick two vertically adjacent coordinates (*i*, *j*) and $(i+1,j)$. If there is a tile *q* placed at $(i+1,j)$, another tile *p* has to be placed at (*i*, *j*), as, otherwise, there is no simply-connected rectangular tiling containing both the tiles at (1, 1) and at $(i+1,j)$. Validity of the tiling also requires identical color codes at the shared edges. On the other hand, if no tile is placed at $(i+1,j)$, a coordinate (*i*, *j*) may be either occupied or empty. The allowed and forbidden combinations are shown in Fig. 5a–d. Formally stated in terms of the decision variables, these considerations are expressed as

13 $$\begin{aligned} \sum _{k \in \mathscr {T}} x_{i,j,k}[c_k^\mathrm{s}=\ell ] - \sum _{k \in \mathscr {T}} x_{i+1, j, k}[c_k^\mathrm{n}=\ell ] \ge 0,\quad \forall (i,j,\ell ) \in \mathscr {H}\setminus \{n_\mathrm{h}\}\times \mathscr {W}\times \mathscr {C}. \end{aligned}$$

Similar arguments hold also for the coordinates (*i*, *j*) and $(i,j+1)$, resulting in the constraints

14 $$\begin{aligned} \sum _{k \in \mathscr {T}} x_{i,j,k}[c_k^\mathrm{e}=\ell ] - \sum _{k \in \mathscr {T}} x_{i, j+1, k}[c_k^\mathrm{w}=\ell ] \ge 0,\quad \forall (i,j,\ell ) \in \mathscr {H} \times \mathscr {W}\setminus \{n_\mathrm{w}\} \times \mathscr {C}. \end{aligned}$$

The allowed and forbidden combinations for this case are shown in Fig. 5e–h.

The developed constraints (11)–(14) enforce simple connectedness; however, they do not guarantee that the resultant tiling will be rectangular. For any 4 adjacent tiles *p*, *q*, *r*, and *s* placed at (*i*, *j*), $(i+1,j)$, $(i,j+1)$, and $(i+1,j+1)$, respectively, these constraints allow for the assemblies shown in Fig. 6. Because the combination 6b cannot appear in any simply-connected rectangular tiling, we must exclude it from the feasible set,

15 $$\begin{aligned} \sum _{k \in \mathscr {T}} x_{i+1,j,k} + \sum _{k \in \mathscr {T}} x_{i,j+1,k} - \sum _{k \in \mathscr {T}} x_{i+1,j+1,k} \le 1, \quad \forall (i,j) \in \mathscr {H} \setminus \{n_\mathrm{h}\} \times \mathscr {W} \setminus \{n_\mathrm{w}\}. \end{aligned}$$

Finally, combining Eqs. (4), (11), (12), (13), (14), and (15) together with an objective function to maximize $|\tilde{\mathscr {B}}_{\max \textrm{rect}}|$ provides us with the binary maximum rectangular valid tiling optimization program

16a $$\begin{aligned} &\max _{\textbf{x}}\,\sum _{i \in \mathscr {H}} \sum _{j \in \mathscr {W}} \sum _{k \in \mathscr {T}} x_{i,j,k} \end{aligned}$$

16b $$\begin{aligned} &\mathrm {s.t.}\,\sum _{k \in \mathscr {T}} x_{i,j,k}[c_k^\mathrm{s}=\ell ] - \sum _{k \in \mathscr {T}} x_{i+1, j, k}[c_k^\mathrm{n}=\ell ] \ge 0,\quad \forall (i,j,\ell ) \in \mathscr {H}\setminus \{n_\mathrm{h}\}\times \mathscr {W}\times \mathscr {C}, \end{aligned}$$

16c $$\begin{aligned}&\sum _{k \in \mathscr {T}} x_{i,j,k}[c_k^\mathrm{e}=\ell ] - \sum _{k \in \mathscr {T}} x_{i, j+1, k}[c_k^\mathrm{w}=\ell ] \ge 0,\quad \forall (i,j,\ell ) \in \mathscr {H}\times \mathscr {W}\setminus \{n_\mathrm{w}\}\times \mathscr {C}, \end{aligned}$$

16d $$\begin{aligned}&\sum _{k \in \mathscr {T}} x_{i+1,j,k} + \sum _{k \in \mathscr {T}} x_{i,j+1,k} - \sum _{k \in \mathscr {T}} x_{i+1,j+1,k} \le 1, \; \forall (i,j) \in \mathscr {H} \setminus \{n_\mathrm{h}\}\times \mathscr {W} \setminus \{n_\mathrm{w}\}, \end{aligned}$$

16e $$\begin{aligned}&\sum _{k \in \mathscr {T}} x_{1,1,k} = 1, \end{aligned}$$

16f $$\begin{aligned}&\sum _{k \in \mathscr {T}} x_{i,j,k} \le 1, \quad \forall (i,j) \in \tilde{\mathscr {A}}\setminus (1,1), \end{aligned}$$

16g $$\begin{aligned}&x_{i,j,k} \in \{0,1\}, \quad \forall (i,j,k) \in \mathscr {H}\times \mathscr {W}\times \mathscr {T}. \end{aligned}$$

In contrast to (10), a feasible solution to the optimization program (16) can be found in a polynomial time, e.g., by tiling the first row or column of the 1D bounded tiling problem. However, finding an optimal solution to (16) is $\mathscr{N}\mathscr{P}$-hard, because the optimization problem (16) is reducible to the decision version (10) by fixing the value of the objective function to $|\tilde{\mathscr {A}} |$, which enforces equalities in (16b), (16c), and (16f), making the constraint (16d) redundant as a consequence.

### Maximum cover

Another option for avoiding the infeasibility of (10) rests in neglecting the requirement of (simple) connectedness, hence allowing for a placement of empty tiles (voids). In this section, we therefore search the maximum cover of $\mathscr {A}$, or equivalently a valid tiling of the (possibly disconnected) domain $\mathscr {B}_{{\max \textrm{cov}}} \subseteq \mathscr {A}$. For the maximum cover formulation, we assume that any two adjacent tiles satisfy the edge-matching constraints of valid tilings, but these are also satisfied by any of the tile-void, void-tile, or void-void combination, where $\sum _{k \in \mathscr {T}} x_{i,j,k} = 0$ for a void located at $(i,j) \in \tilde{\mathscr {A}}$.

Thus, each coordinate (*i*, *j*) is occupied either by a tile or a void, implying that

17 $$\begin{aligned} \sum _{k \in \mathscr {T}} x_{i,j,k} \le 1, \quad \forall (i,j) \in \mathscr {H}\times \mathscr {W}, \end{aligned}$$

and the vertical and horizontal edge matching conditions become

18a $$\begin{aligned} \sum _{k \in \mathscr {T}} x_{i,j,k}[c_k^\mathrm{e}=\ell ] + \sum _{k \in \mathscr {T}} x_{i, j+1, k}[c_k^\mathrm{w} \ne \ell ]&\le 1,\quad \forall (i,j,\ell ) \in \mathscr {H}\times \mathscr {W} \setminus \{n_{\textrm{w}}\}\times \mathscr {C}, \end{aligned}$$

18b $$\begin{aligned} \sum _{k \in \mathscr {T}} x_{i,j,k}[c_k^\mathrm{s}=\ell ] + \sum _{k \in \mathscr {T}} x_{i+1, j, k}[c_k^\mathrm{n}\ne \ell ]&\le 1,\quad \forall (i,j,\ell ) \in \mathscr {H} \setminus \{n_{\textrm{h}}\}\times \mathscr {W}\times \mathscr {C}. \end{aligned}$$

Finally, the combination of Eqs. (17), (18a), (18b) with the objective function to maximize $|\tilde{\mathscr {B}}_{\max \textrm{cov}} |$ leads to the binary optimization problem

19a $$\begin{aligned} \max _{\textbf{x}}\,&\sum _{i \in \mathscr {H}} \sum _{j \in \mathscr {W}} \sum _{k \in \mathscr {T}} x_{i,j,k} \end{aligned}$$

19b $$\begin{aligned} \mathrm {s.t.}\,&\sum _{k \in \mathscr {T}} x_{i,j,k}[c_k^\mathrm{e}=\ell ] + \sum _{k \in \mathscr {T}} x_{i, j+1, k}[c_k^\mathrm{w} \ne \ell ] \le 1,\quad \forall (i,j,\ell ) \in \mathscr {H} \times \mathscr {W} \setminus \{n_{\textrm{w}}\} \times \mathscr {C}, \end{aligned}$$

19c $$\begin{aligned}&\sum _{k \in \mathscr {T}} x_{i,j,k}[c_k^\mathrm{s}=\ell ] + \sum _{k \in \mathscr {T}} x_{i+1, j, k}[c_k^\mathrm{n}\ne \ell ] \le 1,\quad \forall (i,j,\ell ) \in \mathscr {H} \setminus \{n_{\textrm{h}}\} \times \mathscr {W} \times \mathscr {C}, \end{aligned}$$

19d $$\begin{aligned}&\sum _{k \in \mathscr {T}} x_{i,j,k} \le 1, \quad \forall (i,j) \in \mathscr {H} \times \mathscr {W}, \end{aligned}$$

19e $$\begin{aligned}&x_{i,j,k} \in \{0,1\}, \quad \forall (i,j,k) \in \mathscr {H} \times \mathscr {W} \times \mathscr {T}. \end{aligned}$$

The program (19) is trivially $\mathscr{N}\mathscr{P}$-hard: Requiring the objective function (19a) to be at least $|\tilde{\mathscr {A}} |$ implies that

20 $$\begin{aligned} \sum _{k \in \mathscr {T}} x_{i,j,k} = 1, \quad \forall (i,j) \in \mathscr {H} \times \mathscr {W}, \end{aligned}$$

i.e., all positions are occupied by a Wang tile. Moreover, (19b) and (19c) require all adjacent tiles to share the color codes at their common edges. Consequently, the resulting tiling is void-free and valid, and solves the $\mathscr{N}\mathscr{P}$-complete bounded tiling problem.

### Maximum adjacency constraints satisfaction

Because the decision problem (10) also constitutes a specific instance of the constraint satisfaction problem (CSP), another optimization variant comes from the formulation of the max-CSP problem, maximizing the number of satisfied clauses—color matches in our case.

Therefore, for each vertical and horizontal edge we introduce a new variable $h_{i,j}^\mathrm{v} \in \mathbb {R}_{\ge 0}$, where $(i,j) \in \mathscr {H} \times \mathscr {W} \setminus n_\mathrm{w}$, and $h_{i,j}^\mathrm{h} \in \mathbb {R}_{\ge 0}$, with $(i,j) \in \mathscr {H} \setminus n_\mathrm{h} \times \mathscr {W}$, respectively. The adjacency constraints (9) are then relaxed by considering

21a $$\begin{aligned} \left| \sum _{k \in \mathscr {T}} x_{i,j,k}[c_k^\mathrm{s}=\ell ] - \sum _{k \in \mathscr {T}} x_{i+1, j, k}[c_k^\mathrm{n}=\ell ] \right| \le h_{i,j}^\mathrm{h},\quad \forall (i,j, \ell ) \in \mathscr {H}\setminus \{n_\mathrm{h}\} \times \mathscr {W} \times \mathscr {C}, \end{aligned}$$

21b $$\begin{aligned} \left| \sum _{k \in \mathscr {T}} x_{i,j,k}[c_k^\mathrm{e}=\ell ] - \sum _{k \in \mathscr {T}} x_{i, j+1, k}[c_k^\mathrm{w}=\ell ] \right| \le h_{i,j}^\mathrm{v},\quad \forall (i,j,\ell ) \in \mathscr {H} \times \mathscr {W}\setminus \{n_\mathrm{w}\} \times \mathscr {C} \end{aligned}$$

instead. Indeed, if $h_{i,j}^\mathrm{h} = 0$, the edge-matching requirement of the neighboring tiles at (*i*, *j*) and $(i+1,j)$ is satisfied; and it is violated otherwise. Similarly, $h_{i,j}^\mathrm{v} = 0$ guarantees color matches among the tiles at (*i*, *j*) and $(i,j+1)$.

Finally, rewriting absolute values in (21) by two linear inequalities while supplying an objective function to maximize the number of color matches yields the binary optimization problem

22a $$\begin{aligned} \max _{\textbf{x}}\,&\sum _{i\in \mathscr {H}} \sum _{j \in \mathscr {W} \setminus n_\mathrm{w}} \left( 1 - h_{i,j}^\mathrm{v}\right) + \sum _{i\in \mathscr {H} \setminus n_\mathrm{h}} \sum _{j \in \mathscr {W}} \left( 1 - h_{i,j}^\mathrm{h}\right) \end{aligned}$$

22b $$\begin{aligned} \mathrm {s.t.}\,&\sum _{k \in \mathscr {T}} x_{i,j,k}[c_k^\mathrm{s}=\ell ] - \sum _{k \in \mathscr {T}} x_{i+1, j, k}[c_k^\mathrm{n}=\ell ] \le h_{i,j}^\mathrm{h},\;\; \forall (i,j,\ell ) \in \mathscr {H}\setminus \{n_\mathrm{h}\} \times \mathscr {W} \times \mathscr {C}, \end{aligned}$$

22c $$\begin{aligned}&\sum _{k \in \mathscr {T}} x_{i+1, j, k}[c_k^\mathrm{n}=\ell ] -\sum _{k \in \mathscr {T}} x_{i,j,k}[c_k^\mathrm{s}=\ell ] \le h_{i,j}^\mathrm{h},\;\; \forall (i,j,\ell ) \in \mathscr {H}\setminus \{n_\mathrm{h}\} \times \mathscr {W} \times \mathscr {C}, \end{aligned}$$

22d $$\begin{aligned}&\sum _{k \in \mathscr {T}} x_{i,j,k}[c_k^\mathrm{e}=\ell ] - \sum _{k \in \mathscr {T}} x_{i, j+1, k}[c_k^\mathrm{w}=\ell ] \le h_{i,j}^\mathrm{v},\;\; \forall (i,j,\ell ) \in \mathscr {H} \times \mathscr {W}\setminus \{n_\mathrm{w}\} \times \mathscr {C}, \end{aligned}$$

22e $$\begin{aligned}&\sum _{k \in \mathscr {T}} x_{i, j+1, k}[c_k^\mathrm{w}=\ell ] - \sum _{k \in \mathscr {T}} x_{i,j,k}[c_k^\mathrm{e}=\ell ] \le h_{i,j}^\mathrm{v},\;\; \forall (i,j,\ell ) \in \mathscr {H} \times \mathscr {W}\setminus \{n_\mathrm{w}\} \times \mathscr {C}, \end{aligned}$$

22f $$\begin{aligned}&\sum _{k \in \mathscr {T}} x_{i,j,k} = 1, \;\; \forall (i,j) \in \mathscr {H} \times \mathscr {W}, \end{aligned}$$

22g $$\begin{aligned}&x_{i,j,k} \in \{0,1\}, \;\; \forall (i,j,k) \in \mathscr {H} \times \mathscr {W} \times \mathscr {T}, \end{aligned}$$

that is $\mathscr{N}\mathscr{P}$-hard due to the reduction to (10) after setting all $h_{i,j}^\mathrm{v}$ and $h_{i,j}^\mathrm{h}$ to zeros. A feasible solution can be found in a polynomial time by finding valid row/column tilings for each row/column, so that either term $\sum _{i\in \mathscr {H}} \sum _{j \in \mathscr {W} \setminus n_\mathrm{w}} h_{i,j}^\mathrm{v}$ or $\sum _{i\in \mathscr {H} \setminus n_\mathrm{h}} \sum _{j \in \mathscr {W}} h_{i,j}^\mathrm{h}$ equals zero.

### Extensions

Up to now, we have focused solely on the (re)formulations of the bounded tiling problem, searching for *arbitrary* valid tilings. However, some potential applications may require finer control over the resulting tilings. Thus, in this section, we state some simple extensions to enforce tile- and color-based boundary conditions to solve the tile packing problem^14^ and to enforce (variable-sized) periodic boundary conditions.

#### Tile-based boundary conditions

At first, we consider boundary conditions in the form of prescribed tiles. As the simplest one, we enforce the placement of a tile *k* at (*i*, *j*):

23 $$\begin{aligned} x_{i,j,k} = 1, \quad (i,j,k) \in \mathscr {H} \times \mathscr {W} \times \mathscr {T}. \end{aligned}$$

Similarly, we may prevent tile *k* from being placed there:

24 $$\begin{aligned} x_{i,j,k} = 0, \quad (i,j,k) \in \mathscr {H} \times \mathscr {W} \times \mathscr {T}. \end{aligned}$$

Placement of an identical tile at the coordinates $(i,j) \in \tilde{\mathscr {A}}$ and $(p,q) \in \tilde{\mathscr {A}}$ requires

25 $$\begin{aligned} x_{i,j,k} - x_{p,q,k} = 0, \quad \{i,p\} \in \mathscr {H}, \{j,q\} \in \mathscr {W}, \forall k \in \mathscr {T}. \end{aligned}$$

Conversely, different tiles at these coordinates are secured with

26 $$\begin{aligned} x_{i,j,k} + x_{p,q,k} \le 1,\quad \{i,p\} \in \mathscr {H}, \{j,q\} \in \mathscr {W}, \forall k \in \mathscr {T}. \end{aligned}$$

#### Color-based boundary conditions

In addition to the tile-based constraints, we may also enforce specific color codes for individual edges. To do this, the color of the north edge at $(i,j) \in \tilde{\mathscr {A}}$ is set to $\ell$ by

27 $$\begin{aligned} \sum _{k \in T} x_{i,j,k} [c^\mathrm{n}_k = \ell ] = 1, \quad (i,j,\ell ) \in \mathscr {H} \times \mathscr {W} \times \mathscr {C}. \end{aligned}$$

On the contrary, we may prevent this color by requiring

28 $$\begin{aligned} \sum _{k \in T} x_{i,j,k} [c^\mathrm{n}_k = \ell ] = 0, \quad (i,j,\ell ) \in \mathscr {H} \times \mathscr {W} \times \mathscr {C}. \end{aligned}$$

Further, the same color codes at the north edge of $(i,j) \in \tilde{\mathscr {A}}$ and at the west edge of $(p,q) \in \tilde{\mathscr {A}}$ are established with

29 $$\begin{aligned} \sum _{k \in T} x_{i,j,k} [c^\mathrm{n}_k = \ell ] - \sum _{k \in T} x_{p,q,k} [c^\mathrm{w}_k = \ell ] = 0, \quad \{i,p\} \in \mathscr {H}, \{j,q\} \in \mathscr {W}, \forall \ell \in \mathscr {C}, \end{aligned}$$

and a different color with

30 $$\begin{aligned} \sum _{k \in T} x_{i,j,k} [c^\mathrm{n}_k = \ell ] + \sum _{k \in T} x_{p,q,k} [c^\mathrm{w}_k = \ell ] \le 1, \quad \{i,p\} \in \mathscr {H}, \{j,q\} \in \mathscr {W}, \forall \ell \in \mathscr {C}. \end{aligned}$$

#### Periodic tiling

In the domino problem, Wang^1^ investigated the existence of tile sets admitting infinite aperiodic tilings. Here, we consider a similar setting for the finite domain $\mathscr {A}$: examining periodicity through periodic color-based boundary conditions.

We begin with requiring equal coloring at the fixed opposite domain boundaries,

31a $$\begin{aligned}&\sum _{k \in T} x_{1,j,k} [n_k = \ell ] - \sum _{k \in T} x_{n_{\textrm{t},h},j,k} [s_k = \ell ] = 0, \quad \forall (j,\ell ) \in \mathscr {W} \times \mathscr {C}, \end{aligned}$$

31b $$\begin{aligned}&\sum _{k \in T} x_{i,1,k} [w_k = \ell ] - \sum _{k \in T} x_{i,n_{\textrm{t},w},k} [e_k = \ell ] = 0, \quad \forall (i,\ell ) \in \mathscr {H} \times \mathscr {C}. \end{aligned}$$

When adding (31) to the decision problem (10), we thus ask for an existence of a fixed-sized periodic Wang tiling.

In a natural generalization, we ask for an existence of finite-sized periodic Wang tilings, thus relying on the maximum rectangular valid tiling formulation (16). Naturally, the domain size is not known in this case. Therefore, we must consider $\forall (i,j,\ell ) \in \mathscr {H} \times \mathscr {W}\times \mathscr {C}$ constraints of the form

32a $$\begin{aligned} \sum _{k \in \mathscr {T}} x_{i,j,k}[e_k\ne \ell ] + \sum _{k \in \mathscr {T}} x_{i, 1, k}[w_k=\ell ] - \sum _{k \in \mathscr {T}} x_{i,j+1,k} [j<n_{\textrm{t},w}] \le 1, \end{aligned}$$

32b $$\begin{aligned} \sum _{k \in \mathscr {T}} x_{i,j,k}[s_k \ne \ell ] + \sum _{k \in \mathscr {T}} x_{1, j, k}[n_k=\ell ] - \sum _{k \in \mathscr {T}} x_{i+1,j,k}[i<n_{\textrm{t},h}] \le 1. \end{aligned}$$

Here, (32a) prevents a color mismatch of the north edge of $(1,j) \in \tilde{\mathscr {A}}$ and the south edge of $(i,j) \in \mathscr {A}$ iff there is no tile placed at $(i,j+1) \in \tilde{\mathscr {A}}$. Similarly, in the case of (32b), we prevent a color mismatch of the west edge at $(i,1) \in \tilde{\mathscr {A}}$ and the east edge at $(i,j) \in \tilde{\mathscr {A}}$ iff the position $(i+1,j) \in \tilde{\mathscr {A}}$ is empty.

Finally, when adding the constraints (32) to (16, we usually search for the smallest periodic pattern rather than the largest,

33 $$\begin{aligned} \min _{\textbf{x}}\,&\sum _{i \in \mathscr {H}} \sum _{j \in \mathscr {W}} \sum _{k \in \mathscr {T}} x_{i,j,k}. \end{aligned}$$

#### Tile packing problem

Our last extension constitutes the setting of the tile-packing problem^14^: we require each tile to be placed exactly once yet form a fixed-sized valid tiling,

34 $$\begin{aligned} \sum _{i \in \mathscr {H}} \sum _{j \in \mathscr {W}} x_{i,j,k} = 1, \quad \forall k \in \mathscr {T}. \end{aligned}$$

Note here that this extension requires that $|\mathscr {T} |= |\tilde{\mathscr {A}} |$ as, otherwise, no solution exists.

## Heuristic algorithm for the maximum cover tiling problem

In the previous sections, we have introduced several integer programming formulations for the bounded Wang tiling problem and their extensions. Because of the asymptotic complexity of the integer programming formulations, we further develop a simple heuristic algorithm for one of the optimization variants, the maximum cover.

### Maximum row cover tilings

Let us start with revising the decision program (10). In this formulation, neglecting any pair of the constraints (10b, 10d) or (10c, 10e) provides a totally unimodular constraint matrix, recall “Rectangular valid tiling” section. Consequently, such simplified problems are deterministically solvable using the simplex method. Moreover, this setting agrees with the maximum flow problem^61^, as (10d) and (10e) correspond to the flow balances in the source and sink, whereas (10b) and (10c) correspond to the Kirchhoff law equations. Further complexity reduction is possible by recognizing the (shortest) path problem structure, since the source and sink capacities are equal to one, allowing only a single source-to-sink path with positive flow to emerge. Omitting any of these constraint pairs produces valid tilings of (finite) stripes, i.e., of rows or columns. However, the edges shared by the neighboring stripes may not comply with the edge matching rules. Starting with this observation, we first focus on an efficient approach to generate valid tilings of the rows.

**Figure 7:**
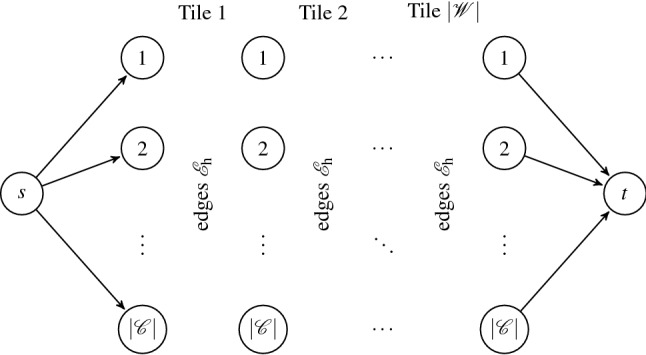
Transducer-based directed acyclic graph for generation of valid row tilings.

As follows from “Notation and preliminaries” section, any valid tiling of a row can be visualized as a $|\mathscr {W} |$-long path in the transducer graph $G_\mathrm{t,h}$, recall “Notation and preliminaries” section. To simplify subsequent developments, we represent the row-tiling problem by a transducer-based directed acyclic graph (DAG) composed of $|\mathscr {W}|+3$ vertex layers. While both the first and the last layer contain only a single vertex (the source *s* and terminal *t*), the intermediate layers include $|\mathscr {C} |$ vertices to represent the vertical (east and west) color codes of the tiles, i.e., the states in the transducer graph. The source vertex is connected to all vertices in the second layer, facilitating an arbitrary coloring of the west edge of the first tile, and, similarly, all the vertices in the penultimate layer are linked to the terminal to allow for all colors in the last east edge. The intermediate layers are bridged with the transducer edges $\mathscr {E}_\mathrm{h}$; see Fig. 7. Consequently, any $s\rightarrow t$ path in the yet-established directed graph forms a valid tiling of the row, and conversely, any valid tiling builds a $s\rightarrow t$ path.

**Figure 8:**
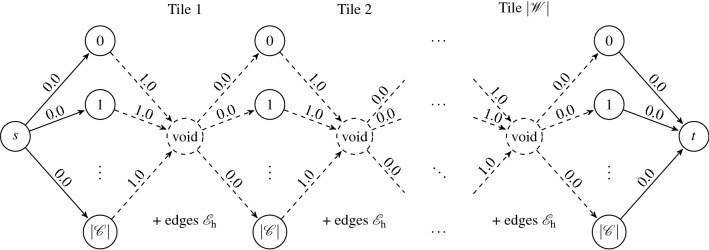
Transducer-based directed acyclic graph for computing the maximum row cover.

However, because such paths do not exist for tile sets that forbid a valid tiling of the row, we also need to incorporate voids. Clearly, we can add “void” tiles as edges that would interconnect the layers, i.e., any two consecutive layers would form a complete bipartite graph. However, such an approach requires adding at most $|\mathscr {W} ||\mathscr {C} |^2$ edges to the graph. Therefore, we add supplementary intermediate layers with a single vertex only, symbolizing the “void” tile type, and connect it to all vertices in the preceding and subsequent layer, see the dashed vertices and edges in Fig. 8. Consequently, we generate at most $2 |\mathscr {W} ||\mathscr {C} |$ new edges altogether.

In addition, we assign unitary costs to the edges incoming to the void vertices and zero costs elsewhere. Hence, the $s\rightarrow t$ path cost is equivalent to the number of voids in the row tiling. Furthermore, because the emergent graph is acyclic and single-sourced, the maximum row-cover tiling is found in $\mathscr {O}(|\mathscr {V} |+ |\mathscr {E} |)$ time using the DAG-shortest-path algorithm^61^, where $\mathscr {V}$ denotes the set of the graph vertices and $\mathscr {E}$ the set of the graph edges. In our case, we have

35a $$\begin{aligned} |\mathscr {V} |&= 2 + (|\mathscr {W}|+ 1)|\mathscr {C} |+ |\mathscr {W}|= 2 + |\mathscr {W}|+ |\mathscr {C}|+ |\mathscr {W}||\mathscr {C}|, \end{aligned}$$

35b $$\begin{aligned} |\mathscr {E} |&= 2|\mathscr {C}|+ 2 |\mathscr {W} ||\mathscr {C} |+ |\mathscr {W} ||\mathscr {T} |. \end{aligned}$$

Thus, the overall asymptotic complexity to generate a maximum row cover tiling evaluates as

36 $$\begin{aligned} \mathscr {O}(|\mathscr {V} |+ |\mathscr {E} |) = \mathscr {O}( 2 + |\mathscr {W}|+ 3 |\mathscr {C}|+ 3 |\mathscr {W} ||\mathscr {C} |+ |\mathscr {W} ||\mathscr {T} |) = \mathscr {O}(|\mathscr {W} ||\mathscr {C} |+ |\mathscr {W} ||\mathscr {T} |). \end{aligned}$$

Interestingly, the running time (but not the asymptotic complexity) of the DAG-shortest-path algorithm can be improved by recognizing that the topological order of the graph vertices—which is required for the DAG-shortest-path algorithm—is known from the graph construction method in advance.

Any path with total cost $c_\mathrm{t}$ contains exactly $c_\mathrm{t}$ voids in the row tiling. Because the shortest path algorithm therefore minimizes the number of voids, it generates the maximum row cover as its output. These considerations are summarized below.

#### Proposition 4.1

The shortest path in the graph in Fig. 8 is equivalent to the maximum row cover.

### Tiling consecutive rows

Assuming already covered rows $i-1$ and $i+1$, e.g., initially by voids, we aim to generate the maximum cover of the *i*-th row. Interestingly, this only requires a minor modification of the graph in Fig. 8.

For this, we first check the north-east compatibility for each tile $k \in \mathscr {T}$ placed at (*i*, *j*). Notice that the compatibility is never violated when the neighbors are voids. For color mismatch cases, we remove the edges denoting these incompatible tiles from the graph.

Assume that the rows $(i-1)$ and $(i+1)$ are voids. Then, clearly, inappropriate tiles at the *i*-th row may prevent the vertically-adjacent positions to be populated by tiles. To limit the appearance of such introduced voids, we include a small penalty of $\epsilon = 1/2 (|\mathscr {W} |+1)^{-1}$ to the tiles that admit a single vertical neighbor only, and $\epsilon = (|\mathscr {W} |+1)^{-1}$ to tiles not admitting any vertical neighbor. Notice that these costs are selected such that, in the worst case, the total penalty due to these void-preventing weights amounts to $|\mathscr {W}|/(|\mathscr {W}|+1)<1$, i.e., the maximum number of tiles is placed even if the void positions forbid any vertical neighbors. Hence, Proposition 4.1 remains satisfied.

Consequently, we can build a simple heuristic algorithm, Algorithm 1, that requires $|\mathscr {H} |$ maximum row-cover iterations, rendering the overall complexity to be $\mathscr {O}(|\tilde{\mathscr {A}} ||\mathscr {C} |+ |\tilde{\mathscr {A}} ||\mathscr {T} |)$.
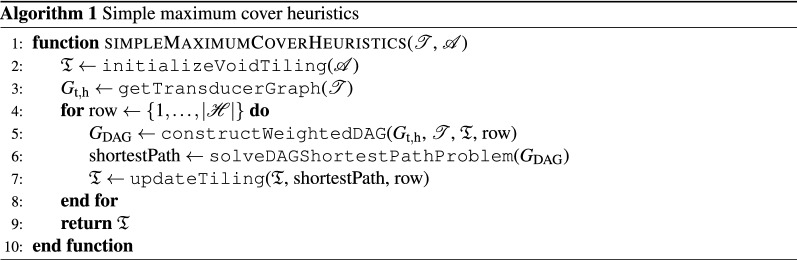


Although Algorithm 1 usually generates relatively large ratio of the number of placed tiles $|\tilde{\mathscr {B}}_\mathrm{cov}|$ to $|\tilde{\mathscr {A}}|$, it probably lacks a guaranteed lower bound. Such bounds can, however, be provided by fairly straightforward modifications introduced next.

### 1/2-approximation algorithm for general tile sets

In this section, we modify Algorithm 1 to maintain the 1/2 approximation ratio. We start with the following observation:

#### Proposition 4.2

Consider the maximum row-cover tiling of the *odd* rows of the initially void domain $\mathscr {A}$ given in “Maximum row cover tilings” section. Then, $|\tilde{\mathscr {B}}_\mathrm{cov}|\ge 1/2 |\tilde{\mathscr {B}}_{\max \textrm{cov}} |$.

#### Proof

Consider that the maximum row-cover problem alone terminates with $|\tilde{\mathscr {B}}_{\max \textrm{rowcov}} |$ tiles. Based on the maximum row-cover property in Proposition 4.1, none of the rows of $\tilde{\mathscr {A}}$ admit a tiling by more than $|\tilde{\mathscr {B}}_{\max \textrm{rowcov}} |$ tiles. Hence, we have $|\tilde{\mathscr {B}}_\mathrm{cov}|\ge \lceil 1/2 |\mathscr {H}|\rceil |\tilde{\mathscr {B}}_{\max \textrm{rowcov}}|$ and $|\tilde{\mathscr {B}}_{\max \textrm{cov}}|\le |\mathscr {H}||\tilde{\mathscr {B}}_{\max \textrm{rowcov}}|$, so that $|\tilde{\mathscr {B}}_\mathrm{cov}|\ge \lceil 1/2|\mathscr {H}|\rceil |\tilde{\mathscr {B}}_{\max \textrm{rowcov}}|\ge 1/2 |\mathscr {H}||\tilde{\mathscr {B}}_{\max \textrm{rowcov}}|\ge 1/2 |\tilde{\mathscr {B}}_{\max \textrm{cov}} |$, where $\lceil \bullet \rceil$ rounds $\bullet$ to the nearest greater or equal integer. $\square$

To exploit Proposition 4.2 in Algorithm 1, we modify the row processing order to $\{1,3,2,5,4\dots \}$. Indeed, then each odd row contains exactly $|\tilde{\mathscr {B}}_{\max \textrm{rowcov}}|$ tiles. Nevertheless, covering the *i*-th (odd) row without acknowledging which tiles are placed in the $(i-2)$-th row may result in an unnecessarily empty $(i-1)$-th row. To avoid such situations, we do not check for compatibility with the $(i-1)$-th row voids, but rather we check using the dual transducer graph with the tiles in the $(i-2)$-th row. For each south color code in the $(i-2)$-th row, we find admissible colors (states) in the dual transducer graph as the states reachable by an edge-long path. Indeed, the reached states are exactly the admissible north colors of compatible tiles in the *i*-th row. For the special case of voids in the $(i-2)$-th row, all color codes are assumed to be compatible. Finally, we penalize the incompatibilities with the cost $\epsilon = 1/2 (|\mathscr {W} |+1)^{-1}$ as before. The final algorithm then reads as Algorithm 2, allowing us to state the following, slightly stronger result:
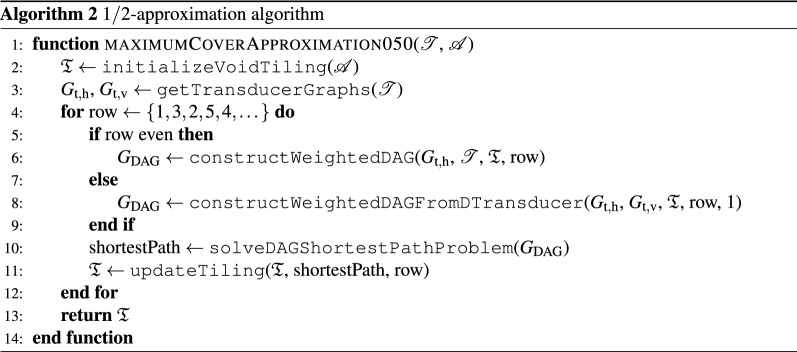

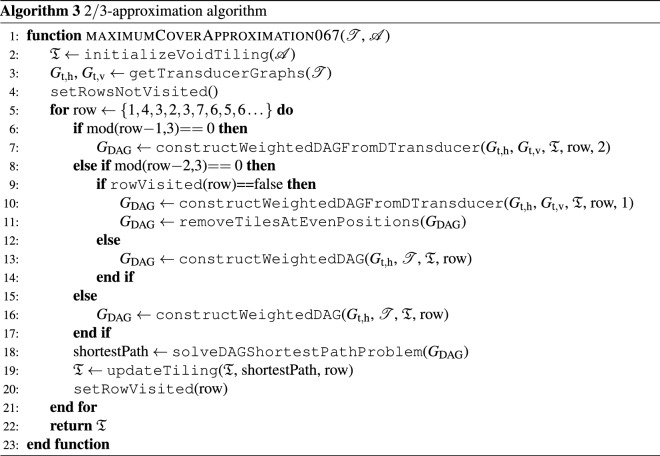


#### Proposition 4.3

Assume a tile set $\mathscr {T}$ with the longest path in its transducer graph $G_{\textrm{t},\textrm{h}}$ of at least 2. Then, Algorithm 2 terminates with $|\tilde{\mathscr {B}}_\mathrm{cov}|\ge 1/2 |\tilde{\mathscr {A}}|$.

#### Proof

When $|\tilde{\mathscr {B}}_{\max \textrm{rowcov}} |= |\mathscr {W}|$, the proof follows directly from Proposition 4.2. For the other cases, the odd rows must contain $|\tilde{\mathscr {B}}_{\max \textrm{rowcov}} |$ tiles due to Proposition 4.1. Because these row-covers are maximal, the sequence of consecutive voids in these rows cannot exceed two, as we could have placed an additional tile otherwise, contradicting with the maximum row-cover property. Moreover, without loss of generality, the cost of the shortest path in the *i*-th row is at most $|\tilde{\mathscr {B}}_{\max \textrm{rowcov}} |+ (|\mathscr {W}|- |\tilde{\mathscr {B}}_{\max \textrm{rowcov}} |) \epsilon$, which occurs when the $(i-2)$-th and *i*-th row have the same tile-void patterns. Because the longest void sequence is at most two and the longest path in $G_{\textrm{t},\textrm{h}}$ is at least two, we can always place tiles to the north of the voids of the *i*-th row. $\square$

### 2/3-approximation algorithm for tilesets with cyclic transducers

Another improvement in the approximation factor of Algorithm 2 is possible for tile sets with all the states in the transducer graphs $G_{\textrm{t},\textrm{h}}$ and $G_{\textrm{t},\textrm{v}}$ being in at least one graph cycle. Notice that this situation occurs for all tile sets that tile the infinite plane.

To this goal, we modify the assignment of costs to graph, and the row processing order to $\{1,4,3,2,3,7,6,5,6,\dots \}$. We begin with (i) tiling the maximum row-cover of the first row. Then, we (ii) find the maximum row-cover of the 4th row such that we penalize possible incompatibilities with the first row based on the dual transducer graph by $\epsilon$. The step (iii) encompasses finding a cover of the 3rd row with penalized incompatibilities with the first row and enforced voids at even positions. Finally, we find the maximum covers for rows 2 and 3. We repeat the procedure for the row numbers iteratively increased by 3, see Algorithm 3. Then, we can make the following statement:

#### Lemma 4.1

Consider that all states in the transducer graphs $G_{\textrm{t},h}$ and $G_{\textrm{t},\textrm{v}}$ are in at least one graph cycle. Then, Algorithm 3 terminates with at least $\frac{2}{3} |\tilde{\mathscr {A}}|$ placed tiles.

#### Proof

Since the tile set allows for valid tiling of the row, the $\{1,4,\;\dots \}$ rows are occupied by exactly $|\mathscr {W} |$ tiles. The $\{3,6,\;\dots \}$ rows are then populated by at least $1/2|\mathscr {W}|$ tiles because each tile from rows $\{4,7,\dots \}$ admits a vertical neighbor. Finally, the $\{2,5,\;\dots \}$ rows contain at least the complement of the number of tiles used in the preceding row, because the tiles in the $\{1,4,6,\dots \}$ row admit a south neighbor. Depending on the number of rows, the algorithm places at least

37 $$\begin{aligned} |\tilde{\mathscr {B}}_{\textrm{cov}} |\ge \min \left\{ |\tilde{\mathscr {A}} |, \frac{3}{4}|\tilde{\mathscr {A}} |, \frac{2}{3}|\tilde{\mathscr {A}} |, \frac{3}{4}|\tilde{\mathscr {A}} |,\frac{7}{10}|\tilde{\mathscr {A}} |,\frac{2}{3}|\tilde{\mathscr {A}} |,\dots \right\} = \frac{2}{3} |\tilde{\mathscr {A}} |\end{aligned}$$

tiles. $$\square$$

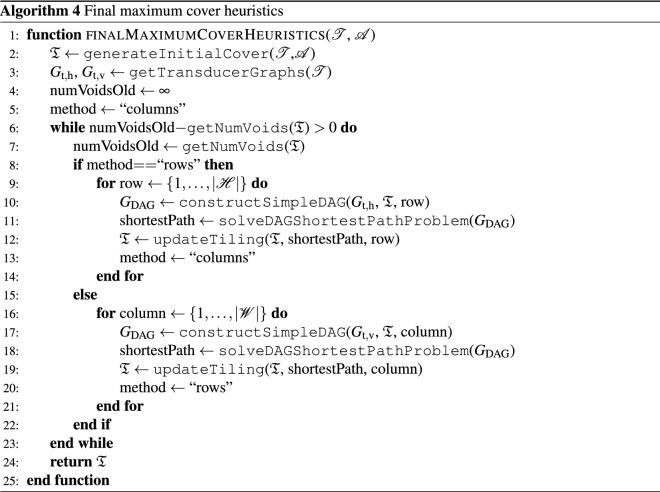



### Iterative improvements

Similarly to finding the maximum row covers, we can search for the maximum cover of columns. When combining these two methods, we end up with our final algorithm that has the $\mathscr {O}(|\tilde{\mathscr {A}} |^2 |\mathscr {C} |+ |\tilde{\mathscr {A}} |^2 |\mathscr {T} |+ |\mathscr {C} |^2)$ complexity and provides the approximation ratios adjustable by algorithm choice (Algorithms 1, 2 or 3) at line 2 of Algorithm 4.

#### Proposition 4.4

Algorithm 4 runs in a polynomial time and terminates in a finite number of steps.

#### Proof

We have already shown that finding a maximum row-cover has $\mathscr {O}(|\mathscr {W} ||\mathscr {C} |+ |\mathscr {W} ||\mathscr {T} |)$ complexity. Further, finding the 2-long paths in the transducer graph possesses the $|\mathscr {C}|^2$ complexity and can be run only once prior to the algorithm main loop. Altogether, Algorithm 3 requires at most $4/3|\mathscr {H}|$ inner iterations so that we have the $\mathscr {O}(|\tilde{\mathscr {A}} ||\mathscr {C} |+ |\tilde{\mathscr {A}} ||\mathscr {T} |+ |\mathscr {C} |^2)$ overall complexity.

Regardless of the method at line 2 of Algorithm 4, the improving loop runs at most $|\tilde{\mathscr {A}} |$ times. Consequently, the algorithm is finite and possesses the $\mathscr {O}(|\tilde{\mathscr {A}} |^2 |\mathscr {C} |+ |\tilde{\mathscr {A}} |^2 |\mathscr {T} |+ |\mathscr {C}|^2)$ complexity. $\square$

## Results

Having developed several exact and heuristic methods, this section is devoted to their numerical examination. We begin with assessing the performance of the integer programming formulations in “Integer programming formulations” section. Then, in “Heuristic algorithms” section, we also relate these results to the outputs of the heuristic algorithms.

Extensions of the integer programs are investigated in subsequent sections. First, we demonstrate the usefulness of the packing constraint by comparing the efficiency of the solution to the tile-packing problem using our method with the times reported by Lagae and Dutré^14^, “Periodic tile packing problem” section. Subsequently, we also present two unexpected discoveries revealed when testing formulations: the Knuth^22^ tile set contains a tile unusable in infinite tilings, “Unusable tile in the Knuth tile set” section, and the Lagae et al.^35^ tile set of 44 corner tiles lacks aperiodicity, “Periodicity of the Lagae corner tile set” section.

We implemented all the methods described above in C++. As the integer programming solver, we used the state-of-the-art optimizer Gurobi 9.5.0^62^ dynamically linked to the compiled binary. Numerical tests were evaluated on a personal laptop running the Ubuntu 18.04 operating system equipped with 24 GB of RAM and Intel$^{\circledR }$ Core$^{\circledR }$ i5-8350U CPU clocked at 1.70GHz.

### Integer programming formulations

In this section, we investigate the performance of all integer programming formulations from “Integer programming formulations” section, i.e., the decision program (10), the maximum rectangular tiling (16), the maximum cover (19), and the maximum adjacency constraint satisfaction problem (22).

**Figure 9:**
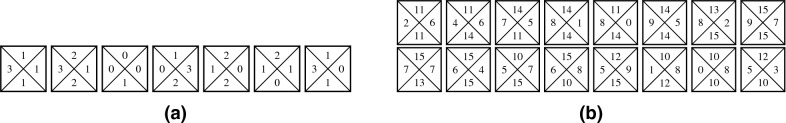
New tile sets (**a**) Finite1 of 7 tiles over 4 colors, and (**b**) Finite2 of 16 tiles over 16 colors used in our algorithmic tests.

We are unaware of any standard sets for bounded tiling problems except for the specific, mostly aperiodic tile sets listed in the literature, recall “Aperiodic tile sets” section. Hence, we consider a set of benchmark problems consisting of five aperiodic tile sets (11 tiles over 4 colors by Jeandel and Rao^33^, 13 tiles over 5 colors by Čulík^29^, 14 tiles over 6 colors by Kari^28^, 16 tiles over 6 colors by Ammann^25^, and 56 tiles over 12 colors by Robinson^24^), two stochastic tile sets introduced in computer graphics (8 tiles over 2 colors by Cohen et al.^9^ and a set of 16 tiles over 4 edge colors by Lagae and Dutré^34^), two periodic tile sets (10 tiles over 4 colors by Wang^19^ and the set of 30 tiles over 17 edge colors by Lagae et al.^35^ and Nurmi^36^). In addition, in Fig. 9, we introduce two tile sets that do not allow for a valid tiling of the infinite domain.

For all these tile sets, we aimed at generating valid tilings sized, respectively, $20\times 20$, $25 \times 25$, and $30 \times 30$. The running time of the Gurobi solver was limited to 300 seconds for the single-threaded mode.

The results shown in Table 1 illustrate that the performance of the decision program (10) surpasses any of the candidate variants. However, it failed to find an existent feasible solution in the time limit four times. In these cases, the output of the optimization problems (16, 19, 22) provided at least some output. Interestingly, the decision problem (10) also was more efficient in the case of proving that the domain $|\mathscr {A}|$ lacks $\mathscr {T}$-tilability.

**Table 1:** Benchmark results. Values marked by an asterisk denote a premature termination of the integer programming solver. The objective function values are equal to the best feasible lower bounds for the individual formulations found by the optimization algorithm. In these formulations, the objective function values denote feasibility, rectangular area, covered area and the number of satisfied adjacency constraints, respectively.

Tile set	Size	Dec. prog. (10)		Max. rect. (16)		Max. cov. (19)		Max. CSP (22)	
		Time (s)	Objective	Time (s)	Objective	Time (s)	Objective	Time (s)	Objective
Aperiodic1 (11/4)^33^	20 × 20	0.111	0	129.897	400	300.053	*398	300.056	*745
	25 × 25	90.810	0	300.070	*150	300.083	*606	300.071	*1135
	30 × 30	300.069	*Infeasible	300.084	*150	300.097	*861	300.089	*1628
Aperiodic2 (13/5)^29^	20 × 20	0.114	0	145.300	400	300.055	*399	300.059	*742
	25 × 25	178.337	0	300.070	*125	300.082	*612	300.078	*1184
	30 × 30	300.069	*Infeasible	300.089	*60	300.098	*876	300.111	*1655
Aperiodic3 (14/6)^28^	20 × 20	275.339	0	181.171	400	300.058	*397	300.058	*752
	25 × 25	300.057	*Infeasible	300.072	*100	300.086	*619	300.086	*1178
	30 × 30	300.073	*Infeasible	300.092	*90	300.107	*863	300.104	*1610
Aperiodic4 (16/6)^25^	20 × 20	0.142	0	171.136	400	176.584	400	71.141	760
	25 × 25	0.196	0	300.063	*100	300.251	*577	300.085	*1030
	30 × 30	0.251	0	300.265	*60	300.132	*794	300.115	*1616
Aperiodic5 (56/12)^24^	20 × 20	0.294	0	300.107	*20	300.214	*350	302.442	*688
	25 × 25	0.440	0	300.155	*25	300.354	*553	300.197	*1055
	30 × 30	0.648	0	300.228	*30	300.434	*795	301.102	*1569
Stochastic1 (8/2)^9^	20 × 20	0.066	0	0.101	400	0.046	400	4.195	760
	25 × 25	0.091	0	0.125	625	0.089	625	5.598	1200
	30 × 30	0.116	0	0.225	900	0.110	900	10.021	1740
Stochastic2 (16/4)^34^	20 × 20	0.114	0	0.107	400	0.129	400	3.226	760
	25 × 25	0.141	0	0.175	625	0.210	625	6.118	1200
	30 × 30	0.183	0	0.217	900	0.283	900	6.846	1740
Periodic1 (10/4)^19^	20 × 20	0.121	0	107.475	400	111.982	400	54.696	760
	25 × 25	0.153	0	274.813	625	300.066	*584	224.734	1200
	30 × 30	0.193	0	300.977	*81	302.606	*824	300.087	*1628
Periodic2 (30/17)^36^	20 × 20	0.236	0	109.860	400	252.700	400	88.721	760
	25 × 25	0.325	0	300.103	*25	300.222	*545	300.150	*1017
	30 × 30	0.473	0	300.158	*30	300.300	*786	300.204	*1521
Finite1 (7/4)	20 × 20	0.066	Infeasible	300.025	*120	300.051	*378	300.076	*725
	25 × 25	0.086	Infeasible	300.038	*125	300.054	*585	300.061	*1108
	30 × 30	0.105	Infeasible	300.046	*108	300.069	*826	300.080	*1628
Finite2 (16/16)	20 × 20	0.100	Infeasible	300.273	*40	300.207	*326	300.077	*684
	25 × 25	0.133	Infeasible	300.067	*50	300.111	*493	300.094	*1029
	30 × 30	0.168	Infeasible	300.084	*30	300.131	*690	300.128	*1525

Comparison of the optimization variants hints that the maximum cover (19) and the maximum adjacency constraint satisfaction (22) problems scale better than the maximum rectangular tiling (16). Indeed, generating any smaller rectangular domain remains $\mathscr{N}\mathscr{P}$-complete, preventing any polynomial-time approximation algorithm to exist. On the other hand, both the formulations (19) and (22) admit simple heuristics, recall “Integer programming formulations” section, allowing the solver to obtain higher-quality feasible solutions faster.

### Heuristic algorithms

Second, we compare the performance of the maximum cover formulation (19) solved with the heuristic Algorithm 4 supplied with three different initial coverings, i.e., based on Algorithms 1, 2 and 3.

Algorithm 4 ran sequentially. In order to limit the dependence of the heuristic algorithm on the ordering of tiles, we randomized the edge order in the directed acyclic graphs. Thus, we evaluated Algorithm 4 100 times for each of the tested option, and listed the best, worst, and mean results in Table 2.

**Table 2:** Numerical tests of the maximum-cover heuristics, Algorithm 4, initialized based on Algorithms 1, 2, and 3. Best mean runs are highlighted in bold. The objective function values in the “min”, “avg”, and “max” columns denote the smallest, average, and the largest areas covered during 100 independent runs of the heuristic algorithms.

Tile set	Size	Algorithm 4 with Algorithm 1				Algorithm 4 with Algorithm 2				Algorithm 4 with Algorithm 3			
		t [s]	Min	Avg	Max	t [s]	Min	Avg	Max	t [s]	Min	Avg	Max
Aperiodic1 (11/4)^33^	20 × 20	0.024	358	**368.99**	380	0.056	342	360.22	372	0.029	334	347.60	360
	25 × 25	0.040	562	**575.38**	586	0.101	543	563.67	582	0.046	524	537.68	547
	30 × 30	0.065	813	**829.66**	843	0.160	792	812.33	835	0.080	758	779.91	798
Aperiodic2 (13/5)^29^	20 × 20	0.043	354	**369.86**	381	0.054	326	359.86	373	0.051	326	353.21	373
	25 × 25	0.065	564	**577.31**	590	0.099	504	564.92	581	0.118	520	557.49	586
	30 × 30	0.123	818	**831.60**	847	0.179	800	817.11	837	0.186	760	806.65	838
Aperiodic3 (14/6)^28^	20 × 20	0.034	362	375.40	386	0.032	353	365.57	381	0.042	355	**378.87**	388
	25 × 25	0.058	564	585.56	604	0.056	548	569.15	597	0.065	562	**592.28**	604
	30 × 30	0.092	813	843.19	857	0.095	799	830.93	860	0.102	827	**855.68**	871
Aperiodic4 (16/6)^25^	20 × 20	0.031	351	**366.09**	381	0.077	293	333.98	351	0.092	281	339.30	368
	25 × 25	0.052	555	**573.19**	591	0.149	469	524.82	549	0.187	442	533.27	562
	30 × 30	0.096	795	**825.44**	860	0.234	666	758.52	785	0.276	743	773.66	802
Aperiodic5 (56/12)^24^	20 × 20	0.054	344	**360.55**	381	0.149	256	341.48	364	0.171	290	332.83	349
	25 × 25	0.110	540	**563.41**	607	0.289	402	527.68	563	0.472	484	529.63	552
	30 × 30	0.147	782	**811.17**	856	0.473	598	782.99	809	0.601	706	759.48	786
Stochastic1 (8/2)^9^	20 × 20	0.014	400	**400.00**	400	0.012	400	**400.00**	400	0.014	400	**400.00**	400
	25 × 25	0.013	625	**625.00**	625	0.014	625	**625.00**	625	0.021	625	**625.00**	625
	30 × 30	0.016	900	**900.00**	900	0.016	900	**900.00**	900	0.019	900	**900.00**	900
Stochastic2 (16/4)^34^	20 × 20	0.013	400	**400.00**	400	0.015	400	**400.00**	400	0.015	400	**400.00**	400
	25 × 25	0.017	625	**625.00**	625	0.017	625	**625.00**	625	0.019	625	**625.00**	625
	30 × 30	0.025	900	**900.00**	900	0.022	900	**900.00**	900	0.026	900	**900.00**	900
Periodic1 (10/4)^19^	20 × 20	0.026	342	**354.82**	374	0.059	325	339.42	353	0.044	323	337.66	346
	25 × 25	0.043	533	**553.13**	573	0.103	507	528.02	546	0.065	512	527.11	537
	30 × 30	0.065	770	**797.26**	830	0.165	745	764.69	784	0.097	729	758.36	770
Periodic2 (30/17)^36^	20 × 20	0.092	366	**382.23**	400	0.419	259	337.18	368	0.346	281	345.72	369
	25 × 25	0.153	559	**595.04**	620	0.808	503	533.30	568	0.649	537	563.63	575
	30 × 30	0.237	825	**854.86**	887	1.259	724	760.08	802	1.131	768	800.74	822
Finite1 (7/4)	20 × 20	0.023	353	360.71	369	0.047	352	**362.98**	376	0.054	348	357.72	366
	25 × 25	0.039	548	**561.15**	570	0.073	348	559.37	580	0.091	539	554.12	566
	30 × 30	0.056	789	807.79	819	0.111	789	**809.48**	830	0.128	791	804.21	826
Finite2 (16/16)	20 × 20	0.065	334	**344.36**	363	0.113	286	331.80	353	0.104	291	328.83	355
	25 × 25	0.101	518	536.20	552	0.198	493	529.24	545	0.140	511	**544.78**	559
	30 × 30	0.191	745	**770.34**	789	0.393	695	761.45	780	0.382	678	750.89	790

From Table 2, it follows that the initialization with the cover from Algorithm 1 is the most efficient for the tested tile sets, both in terms of speed and performance. The remaining two initializations seem to be fairly comparable on average. While for Algorithm 1, at least $82\%$ of tiles were always placed, only more than $60\%$ followed from Algorithm 2. Using Algorithm 3, we obtained at least $70\%$ tile placement.

When comparing Table 1 with Table 2, a few patterns emerge. First, the heuristic algorithm always generates valid tilings if (any of) the stochastic tile sets are used. For aperiodic and periodic tile sets, Gurobi required a considerably longer time to reach feasible solutions of a similar quality, but usually surpassed the developed algorithms in the time limit of 300 s. In the case of Algorithm 1, it can be seen that the resulting covers are very competitive to the outputs of (19) and also obtained in much shorter times.

**Figure 10:**
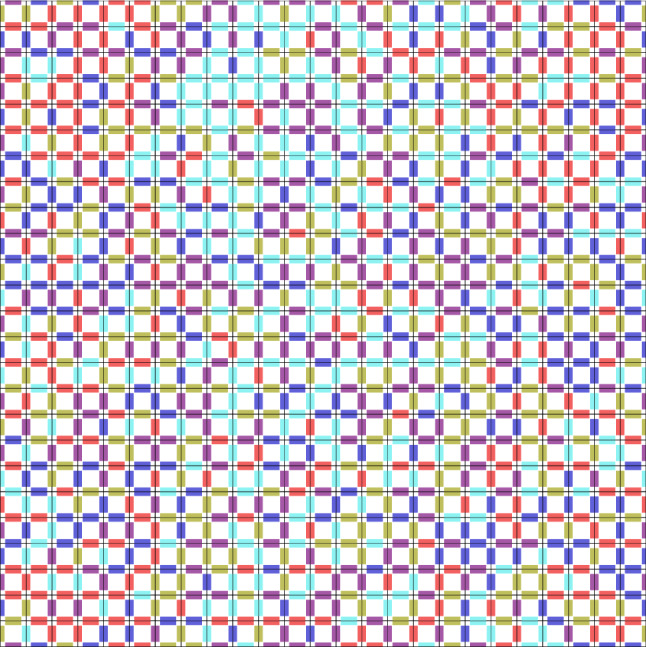
Periodic packing of a complete set of 625 tiles over 5 colors.

### Periodic tile packing problem

As the second numerical example, we consider the periodic tile packing problem investigated in computer graphics applications^14^. Considering a complete edge tile set, Lagae and Dutré searched for a periodic square valid tiling with each tile from the tile set used exactly once. Clearly, such tilings not only contain the entire (textural) information stored in individual tiles but also maintain compatibility with the traditional periodic arrangement.

While Lagae and Dutré^14^ proposed a backtracking-based algorithm to generate periodic packings, we rely here on a solution to the decision program (10) supplemented with the packing (34) and fixed periodicity (31) constraints. The resulting core times spent in the search for a single feasible solution (Table 3) illustrate the higher effectiveness of our method. Consequently, we were able to find a periodic tile packing for the stochastic set of 625 Wang tiles over 5 colors, see Fig. 10.

**Table 3:** Periodic tile packing problem: comparison of core times needed to find a single feasible solution by integer programming (second column) and by the backtracking method (third column) proposed in Lagae and Dutré^14^ to find a feasible solution.

Tile set	Time	Time
	(10, 31, 34)	Lagae and Dutré^14^
Stochastic edge (16/2)	$<1$ s	$<1$ s
Stochastic edge (81/3)	$<1$ s	$<1$ s
Stochastic edge (256/4)	9 s	140 days
Stochastic edge (625/5)	4 days	–

### Unusable tile in the Knuth tile set

One of the oldest aperiodic tile sets, containing 92 tiles over 26 colors, is from Knuth^22^, Exercise 5 in Section 2.3.4.3]. Generating valid tilings from the Knuth tile set using the decision program (10) together with the tile-based boundary conditions, recall “Tile-based boundary conditions” section, led to an unexpected observation that enforced placement of the tile labeled by $\beta U S$ in the Knuth nomenclature^22^ makes the program (10) infeasible under certain circumstances.

After a careful investigation, it indeed turned out that there is not any $2 \times 2$ valid tiling with the $\beta U S$ tile placed at (2, 2). Moreover, there is also not any $4 \times 3$ valid tiling with the $\beta U S$ tile placed at (3, 1). Thus, using the maximum-cover optimization variant (19) and the $\beta U S$ tile enforced at the respective coordinate, there are exactly 31 optimal solutions with the objective function equal to 3, and 498 optimal solutions with the objective function equal to 11.

Consequently, the $\beta U S$ tile can appear only in the strip of at most 3 consecutive infinite columns and does not allow for simply-connected valid tilings of the infinite plane. In a private communication, Knuth confirmed the issue and discovered another 5 tiles that are *unnecessary* but usable in infinite valid tilings, allowing for a possible reduction of the tile set to 86 tiles. For more information, we refer the interested reader to Knuth’s discussion about the reduced tile set^23^, Exercise 221 in 7 Section 7.2.2.1].

### Periodicity of the Lagae corner tile set

Analogously to the Wang tiles, with the connectivity information stored in the edges, Lagae and Dutré^34^ introduced *corner tiles* with colored corners. As Wang^19^ noted in 1975, these formalisms are interchangeable if the (infinite) domino problem is considered, because every set of Wang tiles can be represented by sets of corner tiles with greater or equal cardinality^35^. However, corner tiles avoid the so-called corner problem of Wang tiles in computer graphics^34^, motivating Lagae et al.^35^ to develop conversion methods for transforming Wang tiles to corner tiles, and vice versa. A direct product of these conversions are aperiodic tile sets of corner tiles^35^.

Two of these methods, called horizontal and vertical translations, were used to convert the Ammann set of 16 Wang tiles over 6 colors^25^ to the set of 44 corner tiles over 6 colors, and the resulting isomorphic corner tile sets were claimed aperiodic^35^. In 2016, Nurmi^36^ noticed that, in this set, 14 tiles are unusable in infinite valid tilings, and reduced the corner tile set to 30 tiles over 6 colors. Quite surprisingly, neither Lagae et. al. nor Nurmi recognized that the tile set forms a torus, and is therefore periodic, as we show next.

**Figure 11:**
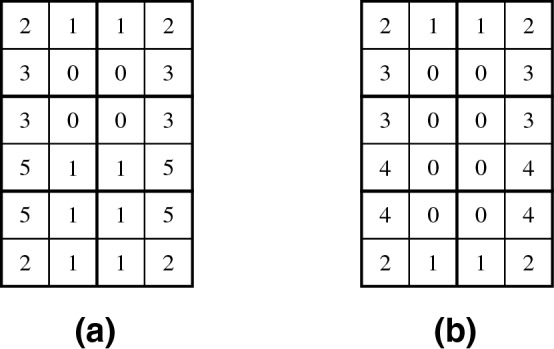
Rectangular periodic valid tilings. Translating a $2\times 3$ rectangle over the infinite valid tiling generated from (**a**) or (**b**) leads to 6 different periodic patterns of the same size. Consequently, the tile set allows for 12 periodic rectangles of the size $2\times 3$.

Instead of developing a new formulation for another type of tiles, we first notice that corner tiles are actually a subset of Wang tiles, and therefore every set of corner tiles can be represented by a set of Wang tiles with the same cardinality, see Appendix. For these tiles, we solve the rectangular tiling formulation (16) with periodic boundary conditions (32) and an objective function to find the smallest tiling (33). As its output, we receive the optimal value of 6 and 12 optimal periodic rectangular tilings of the size $2 \times 3$. Not surprisingly, all these solutions follow from only two periodic patterns shown in Fig. 11 by translations over the infinite plane.

Having revealed the smallest periodic solutions, it remains to be shown why the Lagae conversion methods failed. Lagae et al.^35^ mentioned that their methods lack bijectiveness in general but they assumed it was not the case here. Therefore, we believe it is useful to state the conditions under which the methods are bijective and show that they are not satisfied for the Ammann tile set.

#### Lemma 5.1

The horizontal translation method from Lagae et al.^35^ is bijective iff the dual transducer graph $G_\mathrm{T,v}$ of the input tile set $\mathscr {T}$ does not contain any parallel arcs.

#### Proof

The horizontal translation method is formally a mapping $\mathscr {T} \times \mathscr {T} \mapsto \mathscr {T}_\mathrm{corner}$ that generates $\forall (p,q) \in \mathscr {T}^2: c_p^\mathrm{e} = c_q^\mathrm{w}$ a corner tile $(c_p^\mathrm{n}, c_p^\mathrm{s}, c_q^\mathrm{s}, c_q^\mathrm{n})$. To be bijective, the cardinality of the output needs to be equal to the cardinality of the input, and the mapping has to produce unique output for each input. Consequently, all the tiles $p \in \mathscr {T}$ in the original tile set must be uniquely determined by $c_p^\mathrm{n}$ and $c_p^\mathrm{s}$, as the color codes of the vertical edges of $\mathscr {T}$ are avoided in the construction of $\mathscr {T}_\mathrm{corner}$.

Let us now consider that the dual transducer graph contains a parallel arc connecting the state $c^\mathrm{n}$ with $c^\mathrm{s}$. Then, there may exist two tiles colored by $(c^\mathrm{n}, c_p^\mathrm{w}, c^\mathrm{s}, c_p^\mathrm{e})$ and $(c^\mathrm{n}, c_q^\mathrm{w}, c^\mathrm{s}, c_q^\mathrm{e})$ that are indistinguishable in $\mathscr {T}_\mathrm{corner}$, which contradicts the bijection. For the other option, if the transducer graph does not contain any parallel arcs, then each $c_q^\mathrm{n}$, $c_q^\mathrm{s}$ identifies with a single arc labeled by $c_q^\mathrm{w} \vert c_q^\mathrm{e}$, i.e., with a single tile, which completes the proof. $\square$

Rotating the tile set by 90 degrees, the arguments in Lemma 5.1 provide us with the conditions for the bijectiveness of the vertical translation method:

#### Lemma 5.2

The vertical translation method of Lagae et al.^35^ is bijective iff the transducer graph $G_\mathrm{T,h}$ of the input tile set $\mathscr {T}$ does not contain any parallel arcs.

**Figure 12:**
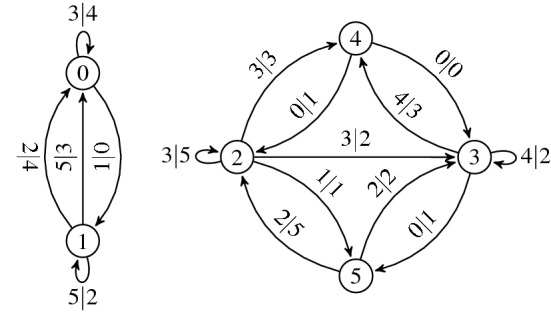
Transducer graph of the Ammann set of 16 Wang tiles over 6 colors.

For the Ammann tile set, we obtain the transducer graph $G_\mathrm{T,h} = G_\mathrm{T,v}$ shown in Fig. 12. Clearly, there exist parallel arcs $1 \rightarrow 0$. Moreover, using the same approach, we can show that the horizontal translation method also fails for the Robinson tile set of 24 tiles over 24 colors^25^, contrary to the claims in^35^, and the corresponding corner tile set is also periodic.

## Conclusions

In this contribution, we investigated methods for generating bounded Wang tilings for arbitrary tile sets. To this goal, we developed four binary linear programming formulations, namely decision (10), maximum-cover (19), maximum adjacency constraint satisfaction (22), and maximum rectangular tiling (16) variants. We supplemented them with extensions for controlling individual tiles and their colors and variable-sized tiling periodicity constraints. The second part of the manuscript was devoted to developing efficient heuristic approximation algorithms for the maximum-cover integer program variant, one maintaining a 1/2 approximation ratio for arbitrary tile sets and another a 2/3 ratio for tile sets with cyclic transducers.

For readers’ convenience, we summarize the outputs of this study as follows:

1. Based on the numerical testing on a collection of 11 tile sets, the decision program (10) is the most efficient. However, when a time limit is imposed or if the tile set does not allow for valid tiling of the entire domain, then the maximum cover (19) and maximum adjacency constraint satisfaction problems (22) appear to be similarly efficient.
2. The maximum rectangular tiling (16) formulation exhibits the worst scalability.
3. The formulation (10) supplemented with the packing constraint (34) maintains a better solution efficiency for the Wang tile packing problem than the Lagae and Dutré^14^ backtracking approach.
4. The integer programming formulations allow to disprove theoretical results in Wang tilings. We illustrated this by
  - revealing a tile in the Knuth^22^ tile set that is unusable in two-way infinite tilings,
  - proving that the Lagae et al.^35^ tile set of corner tiles lacks aperiodicity. We also included an explanation for why the tile set construction method failed.
5. Among the three proposed heuristic algorithms, the setup of Algorithm 4 initialized with the cover generated by Algorithm 1 produced the best results on average. Such algorithm was faster and provided competitive results with the Gurobi software running for 300s.

Having summarized our contributions, we believe that this work has not only introduced new methods that can possibly be applied to materials engineering, but also a simple and quite extensible framework to verify theoretical results on Wang tilings.

## Supplementary Information

Supplementary Information.

## Data Availability

Source code available at: https://gitlab.com/tyburec/tilopt.

## References

[1] Wang, H. Dominoes and the AEA case of the decision problem. In *Symposium on Mathematical Theory of Automata* 23–55 (1963).

[2] Wang H (1961). Proving theorems by pattern recognition-II. Bell Syst. Tech. J..

[3] Berger R (1966). The undecidability of the domino problem. Mem. Am. Math. Soc..

[4] Turing AM (1937). On computable numbers, with an application to the Entscheidungsproblem. Proc. Lond. Math. Soc..

[5] Davis M (1958). Computability & Unsolvability. Dover Books on Computer Science Series.

[6] Kahr AS, Moore EF, Wang H (1962). Entscheidungsproblem reduced to the $$\forall \exists \forall$$ case. Proc. Natl. Acad. Sci..

[7] Lewis HR (1978). Complexity of Solvable Cases of the Decision Problem for the Predicate Calculus.

[8] Lewis H, Papimitriou C (1981). Elements of the Theory of Computation. Prentice-Hall Software Series.

[9] Cohen MF, Shade J, Hiller S, Deussen O (2003). Wang tiles for image and texture generation. ACM Trans. Graph..

[10] Derouet-Jourdan A, Kaji S, Mizoguchi Y (2019). A linear algorithm for brick Wang tiling. Jpn. J. Ind. Appl. Math..

[11] Lagae A, Dutré P (2005). A procedural object distribution function. ACM Trans. Graph..

[12] Ollinger, N. Two-by-two substitution systems and the undecidability of the domino problem. In *Logic and Theory of Algorithms* 476–485 (Springer, 2008). 10.1007/978-3-540-69407-6_51

[13] Kovalsky SZ, Glasner D, Basri R (2015). A global approach for solving edge-matching puzzles. SIAM J. Imaging Sci..

[14] Lagae A, Dutré P (2007). The tile packing problem. Geombinatorics.

[15] Rui Yu, C. R. & Agapito, L. Solving jigsaw puzzles with linear programming. In *Proceedings of the British Machine Vision Conference (BMVC)* (eds Wilson, R. C., Hancock, E. R. & Smith, W. A. P.) 139.1–139.12 (BMVA Press, 2016). 10.5244/C.30.139.

[16] Salassa, F., Vancroonenburg, W., Wauters, T., Della Croce, F. & Berghe, G. V. MILP and max-clique based heuristics for the Eternity II puzzle (2017). arXiv:1709.00252.

[17] Garvie MR, Burkardt J (2022). A parallelizable integer linear programming approach for tiling finite regions of the plane with polyominoes. Algorithms.

[18] Berger, R. *The Undecidability of the Domino Problem*. Ph.D. thesis, Harvard University (1964).

[19] Wang H (1975). Notes on a class of tiling problems. Fundam. Math..

[20] Robinson RM (1967). Seven polygons which permit only nonperiodic tilings of the plane. Not. Am. Math. Soc..

[21] Poizat B (1980). Une théorie finiement axiomatisable et superstable. Groupe d’étude des théories stables.

[22] Knuth DE (1968). The Art of Computer Programming, Volume 1: Fundamental Algorithms.

[23] Knuth DE (2018). The Art of Computer Programming, Volume 4B, Fascicle 5: The: Mathematical Preliminaries Redux; Backtracking; Dancing Links.

[24] Robinson RM (1971). Undecidability and nonperiodicity for tilings of the plane. Invent. Math..

[25] Grünbaum B, Shephard GC (2016). Tilings and Patterns.

[26] Robinson RM (1978). Undecidable tiling problems in the hyperbolic plane. Invent. Math..

[27] Senechal M (1996). Quasicrystals and Geometry.

[28] Kari J (1996). A small aperiodic set of Wang tiles. Discrete Math..

[29] Čulík K (1996). An aperiodic set of 13 Wang tiles. Discrete Math..

[30] Kari J, Papasoglu P (1999). Deterministic aperiodic tile sets. Geom. Funct. Anal..

[31] Labbé S (2019). A self-similar aperiodic set of 19 Wang tiles. Geom. Dedicata.

[32] Labbé, S. & Lepšová, J. A numeration system for Fibonacci-like Wang shifts. In *Lecture Notes in Computer Science* 104–116 (Springer International Publishing, 2021). 10.1007/978-3-030-85088-3_9.

[33] Jeandel E, Rao M (2021). An aperiodic set of 11 Wang tiles. Adv. Comb..

[34] Lagae A, Dutré P (2006). An alternative for Wang tiles: Colored edges versus colored corners. ACM Trans. Graph..

[35] Lagae, A., Kari, J. & Dutré, P. Aperiodic sets of square tiles with colored corners. Report CW (2006).

[36] Nurmi, T. From checkerboard to cloverfield: Using Wang tiles in seamless non-periodic patterns. In *Bridges Finland Conference Proceedings* (2016).

[37] Kari J (1990). Reversibility of 2D cellular automata is undecidable. Phys. D: Nonlinear Phenom..

[38] Conway J, Lagarias J (1990). Tiling with polyominoes and combinatorial group theory. J. Comb. Theory Ser. A.

[39] Mozes S (1989). Tilings, substitution systems and dynamical systems generated by them. J. d’Analyse Mathématique.

[40] Stam, J. *Aperiodic Texture Mapping*. Technical report R046 (European Research Consortium for Informatics and Mathematics, 1997).

[41] Liu X, Li C, Lu L, Deussen O, Tu C (2022). Fabricable multi-scale Wang tiles. Comput. Graph. Forum.

[42] Hiller, S., Deussen, O. & Keller, A. Tiled blue noise samples. In *Proceedings of the Vision Modeling and Visualization Conference* 265–272 (Stuttgart, Germany, 2001).

[43] Radin C (1987). Low temperature and the origin of crystalline symmetry. Int. J. Mod. Phys..

[44] Winfree E, Liu F, Wenzler LA, Seeman NC (1998). Design and self-assembly of two-dimensional DNA crystals. Nature.

[45] Seeman NC, Mao C, LaBean TH, Reif JH (2000). Logical computation using algorithmic self-assembly of DNA triple-crossover molecules. Nature.

[46] Novák J, Kučerová A, Zeman J (2012). Compressing random microstructures via stochastic Wang tilings. Phys. Rev. E.

[47] Doškář M, Novák J, Zeman J (2014). Aperiodic compression and reconstruction of real-world material systems based on Wang tiles. Phys. Rev. E.

[48] Braides A, Riey G, Solci M (2009). Homogenization of Penrose tilings. C. R. Math..

[49] Doškář M, Novák J (2016). A jigsaw puzzle framework for homogenization of high porosity foams. Comput. Struct..

[50] Doškář M, Zeman J, Rypl D, Novák J (2020). Level-set based design of Wang tiles for modelling complex microstructures. Comput. Des..

[51] Tyburec M, Zeman J, Doškář M, Kružík M, Lepš M (2020). Modular-topology optimization with Wang tilings: An application to truss structures. Struct. Multidiscip. Optim..

[52] Tyburec M, Doškář M, Zeman J, Kružík M (2022). Modular-topology optimization of structures and mechanisms with free material design and clustering. Comput. Methods Appl. Mech. Eng..

[53] Jílek M, Somr M, Kulich M, Zeman J, Přeučil L (2021). Towards a passive self-assembling macroscale multi-robot system. IEEE Robot. Autom. Lett..

[54] Jilek M (2022). Self-stabilizing self-assembly. IEEE Robot. Autom. Lett..

[55] Doškář M, Zeman J, Jarušková D, Novák J (2018). Wang tiling aided statistical determination of the Representative Volume Element size of random heterogeneous materials. Eur. J. Mech. A/Solids.

[56] Coulais C, Teomy E, de Reus K, Shokef Y, van Hecke M (2016). Combinatorial design of textured mechanical metamaterials. Nature.

[57] Yang W, Liu Q, Gao Z, Yue Z, Xu B (2018). Theoretical search for heterogeneously architected 2D structures. Proc. Natl. Acad. Sci..

[58] Nežerka V (2018). A jigsaw puzzle metamaterial concept. Compos. Struct..

[59] Yasuda H (2021). Mechanical computing. Nature.

[60] Knuth DE (1992). Two notes on notation. Am. Math. Mon..

[61] Korte B, Vygen J (2006). Combinatorial Optimization.

[62] Gurobi Optimization, LLC. Gurobi Optimizer Reference Manual (2022). http://gurobi.com.

