# Supplementary material for: Bounded Wang tilings with integer programming and graph-based heuristics
Source: Sci Rep. 2023 Mar 24;13:4865. doi: 10.1038/s41598-023-31786-3 (PMC10039085; doi:10.1038/s41598-023-31786-3)
Supplement: Supplementary file 1 — Supplementary Information. [file 41598_2023_31786_MOESM1_ESM.pdf]

A Corner tiles represented as Wang tiles

Each corner tile in the corner tile set is defined by a quadruple of color codes $(c_k^{\mathrm{nw}}, c_k^{\mathrm{sw}}, c_k^{\mathrm{se}}, c_k^{\mathrm{ne}})$, with c_k^{nw}, c_k^{sw}, c_k^{se}, and c_k^{ne} denoting the colors of the northwest, southwest, southeast, and northeast corner of the k-th tile. Similarly to Wang tiles, corner tiles are assembled such that the color codes at the adjoining corners match.

This is, however, also maintained if we denote their edges by labels in the form of tuples of the corner codes, $(c_k^{\mathrm{nw}}, c_k^{\mathrm{ne}})$, $(c_k^{\mathrm{nw}}, c_k^{\mathrm{sw}})$, $(c_k^{\mathrm{sw}}, c_k^{\mathrm{se}})$, and $(c_k^{\mathrm{ne}}, c_k^{\mathrm{se}})$, each of which denotes a single edge label of the north, west, south, and east edge, respectively. Consequently, we can compute unique color codes as

$$c_k^{\mathrm{n}} = c_k^{\mathrm{nw}} + c_k^{\mathrm{ne}} n_{\mathrm{vc}}, \tag{1a}$$

$$c_k^{\mathrm{w}} = c_k^{\mathrm{nw}} + c_k^{\mathrm{sw}} n_{\mathrm{vc}}, \tag{1b}$$

$$c_k^{\mathrm{s}} = c_k^{\mathrm{sw}} + c_k^{\mathrm{se}} n_{\mathrm{vc}}, \tag{1c}$$

$$c_k^{\mathrm{e}} = c_k^{\mathrm{ne}} + c_k^{\mathrm{se}} n_{\mathrm{vc}}, \tag{1d}$$

where n_{vc} stands for the number of colors used in the corner tile set. Graphical illustration of the corner-edge tile equivalence is shown in Fig. 1.

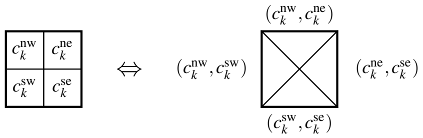

Figure 1. A corner tile expressed using edge formalism.
